# Loose-patch clamp analysis applied to voltage-gated ionic currents following pharmacological ryanodine receptor modulation in murine hippocampal cornu ammonis-1 pyramidal neurons

**DOI:** 10.3389/fphys.2024.1359560

**Published:** 2024-04-24

**Authors:** Federico Bertagna, Shiraz Ahmad, Rebecca Lewis, S. Ravi P. Silva, Johnjoe McFadden, Christopher L.-H. Huang, Hugh R. Matthews, Kamalan Jeevaratnam

**Affiliations:** ^1^ Leverhulme Quantum Biology Doctoral Training Centre, University of Surrey, Guildford, United Kingdom; ^2^ School of Veterinary Medicine, Faculty of Health and Medical Sciences, University of Surrey, Guildford, United Kingdom; ^3^ Advanced Technology Institute, University of Surrey, Guildford, United Kingdom; ^4^ School of Biosciences and Medicine, Faculty of Health and Medical Sciences, University of Surrey, Guildford, United Kingdom; ^5^ Physiological Laboratory, University of Cambridge, Cambridge, United Kingdom; ^6^ Department of Biochemistry, University of Cambridge, Cambridge, United Kingdom

**Keywords:** voltage-gated channels, hippocampal pyramidal neurons, ryanodine receptors, caffeine, dantrolene, cyclopiazonic acid

## Abstract

**Introduction:**

The loose-patch clamp technique was first developed and used in native amphibian skeletal muscle (SkM), offering useful features complementing conventional sharp micro-electrode, gap, or conventional patch voltage clamping. It demonstrated the feedback effects of pharmacological modification of ryanodine receptor (RyR)-mediated Ca^2+^ release on the Na^+^ channel (Nav1.4) currents, initiating excitation–contraction coupling in native murine SkM. The effects of the further RyR and Ca^2+^-ATPase (SERCA) antagonists, dantrolene and cyclopiazonic acid (CPA), additionally implicated background tubular-sarcoplasmic Ca^2+^ domains in these actions.

**Materials and methods:**

We extend the loose-patch clamp approach to ion current measurements in murine hippocampal brain slice cornu ammonis-1 (CA1) pyramidal neurons. We explored the effects on Na^+^ currents of pharmacologically manipulating RyR and SERCA-mediated intracellular store Ca^2+^ release and reuptake. We adopted protocols previously applied to native skeletal muscle. These demonstrated Ca^2+^-mediated feedback effects on the Na^+^ channel function.

**Results:**

Experiments applying depolarizing 15 ms duration loose-patch clamp steps to test voltages ranging from −40 to 120 mV positive to the resting membrane potential demonstrated that 0.5 mM caffeine decreased inward current amplitudes, agreeing with the previous SkM findings. It also decreased transient but not prolonged outward current amplitudes. However, 2 mM caffeine affected neither inward nor transient outward but increased prolonged outward currents, in contrast to its increasing inward currents in SkM. Furthermore, similarly and in contrast to previous SkM findings, both dantrolene (10 μM) and CPA (1 μM) pre-administration left both inward and outward currents unchanged. Nevertheless, dantrolene pretreatment still abrogated the effects of subsequent 0.5- and 2-mM caffeine challenges on both inward and outward currents. Finally, CPA abrogated the effects of 0.5 mM caffeine on both inward and outward currents, but with 2 mM caffeine, inward and transient outward currents were unchanged, but sustained outward currents increased.

**Conclusion:**

We, thus, extend loose-patch clamping to establish pharmacological properties of murine CA1 pyramidal neurons and their similarities and contrasts with SkM. Here, evoked though not background Ca^2+^-store release influenced Nav and Kv excitation, consistent with smaller contributions of background store Ca^2+^ release to resting [Ca^2+^]. This potential non-canonical mechanism could modulate neuronal membrane excitability or cellular firing rates.

## 1 Introduction

The loose-patch clamp method was first introduced to study surface membrane ionic currents in relatively large-diameter amphibian skeletal muscle (SkM) fibers (Stühmer et al., 1983). It used pipettes with relatively large tip diameters, compared to those used in conventional patch clamp, on native *in situ* muscle fibers without requiring enzyme pre-treatment or membrane rupture. The larger pipette areas meant that the pipettes did not form the tight seal made by conventional patch clamps electrodes with the cell membrane. This permits significant current to pass through the lower resulting seal resistances between the membrane and pipette tip. The latter is compensated for by using specific recording and current delivery electronics distinct from those used in conventional tight patch clamping. This can deliver larger compensation currents. Nevertheless, this approach permits repeated electrode applications and withdrawals. Measurements can be made from the same or successive membrane patches on the same cell. The procedure avoids membrane disruption, potentially altering intracellular content. These advantages have proven valuable in investigations of channel localization (Almers et al., 1983) and lateral mobility (Stühmer and Almers, 1982; Roberts et al., 1986). This reversibility of the loose-patch seal additionally allows pair-wise comparisons from the same patch (Stühmer et al., 1983) before and following experimental maneuvers involving the application or withdrawal of pharmacological or osmotic agents (Chin et al., 2004). This overcomes problems of variability between patches or cells (Liu et al., 2021). It also permits studies in successively different cells using the same electrode with consistent geometric and electrical properties (Milton and Caldwell, 1990; Roberts and Almers, 1992). These advantages have been used in extensions of this technique in murine cardiac muscle (Valli et al., 2018a; Ahmad et al., 2019).

The latter advantages proved useful in pharmacological studies of the feedback effects of ryanodine receptor, RyR1- or RyR2-mediated intracellular store Ca^2+^ release upon skeletal or cardiac muscle Na^+^ channel, Nav1.4 or Nav1.5, function in intact *in situ* native loose-patch clamped murine skeletal and cardiac myocytes. These led to discoveries of mechanisms by which the Ca^2+^ signaling initiated by feedforward, excitation–contraction coupling exerts feedback effects on Nav excitability (Salvage et al., 2021; Salvage et al., 2023).

Such studies investigated the effects of RyR-specific agonists and antagonists expected to alter rates of Ca^2+^ release from their sarcoplasmic reticular (SR) stores relative to background release levels. These properties were demonstrated by spectrofluometric cytosolic [Ca^2+^]_i_ measurements in resting mammalian murine skeletal muscle SR or fiber preparations (Fryer and Neering, 1989; Pagala and Taylor, 1998). Here, the direct RyR modulator caffeine (Herrmann-Frank et al., 1999) at 0.5 mM persistently (3–10 min) increased [Ca^2+^]_i_ (to ∼300 nM) from its resting concentrations (typically ∼106 ± 2 nM: (Fryer and Neering, 1989; Head, 1993; Dettbarn et al., 1994). Contrastingly, higher, ≥1.0 mM, caffeine challenge induced early [Ca^2+^]_i_ peaks followed by persistent below-resting [Ca^2+^]_i_ reductions within 80–90 s (Dettbarn et al., 1994; Pagala and Taylor, 1998). The latter was attributed to Ca^2+^-induced sarcoplasmic reticular Ca^2+^ transporter (SERCA) activation, followed by a sustained slow (∼seconds) RyR inactivation, reducing channel open probabilities. This inactivation property was independently demonstrated in RyR1s reconstituted in lipid bilayers exposed to steady [Ca^2+^]_i_ at levels similarly initially activating RyR (∼10–100 µM) (Laver and Curtis, 1996). The same 0.5 mM and 2 mM caffeine concentrations, respectively, decreased and increased peak inward current. Conversely, the RyR1 inhibitor (Zhao et al., 2001) and muscle-relaxant (Ellis et al., 1973) dantrolene (10 μM) and sarcoplasmic reticular (SR) Ca^2+^ depletion produced by the SERCA inhibitor cyclopiazonic acid (CPA; 10 μM) (Matthews et al., 2019; Sarbjit-Singh et al., 2020; Liu et al., 2021) increased peak inward current and abrogated caffeine’s effects.

These findings identified caffeine as increasing and dantrolene and CPA as decreasing RyR1-mediated SR store Ca^2+^ release into a T-SR Ca^2+^ domain (Bardsley et al., 2021) with corresponding effects on Nav activation. Concordant findings emerged in reports from murine atrial and ventricular cardiomyocytes (Valli et al., 2018a; Ahmad et al., 2019). These findings were consistent with molecular structural data bearing on Ca^2+^ action, either direct or through Ca^2+^-calmodulin, in the region of the Nav1.4 or Nav1.5 C-terminal domains (Salvage et al., 2021).

Intracellular Ca^2+^ signaling also operates in central nervous system (CNS) neurons, modulating diverse physiological processes, particularly cell excitability (Berridge, 1998). In hippocampal cornu ammonis-1 (CA1) pyramidal neurons, it is implicated in age-related cognitive decline through altered synaptic plasticity and neuronal excitability (Foster, 2007; Kumar et al., 2009; Burke and Barnes, 2010; Oh et al., 2010), impaired neurogenesis in experimental Alzheimer’s disease models (Mattson and Chan, 2003), and toxic effects, potentially leading to cell death at high concentrations (Stanika et al., 2012). In common with striated muscle, neurons maintain low background intracellular [Ca^2+^]_i_ ∼100 nM levels (Xu et al., 2014) through cytosolic exchanges with the extracellular environment and intracellular, mitochondrial, and endoplasmic reticular (ER) reservoirs. In striated SkM or cardiac muscle, Nav1.4 or Nav1.5-mediated action potential generation (Adrian and Peachey, 1973) increases [Ca^2+^]_i_ through RyR1- or RyR2-mediated intracellular SR Ca^2+^ store release. This involves direct, allosteric, or extracellular Ca^2+^ entry-dependent coupling of transitions in the tubular membrane, voltage-sensing, or Ca^2+^-conducting Cav1.1 or Cav1.2 channels, to the activation of RyR1 or RyR2 SR Ca^2+^ release channels, respectively (Huang et al., 2011). These interactions occur at triad or dyad junctions (Martin et al., 2003) between surface tubular and SR membranes (Chawla et al., 2001) that may form Ca^2+^ domains within their enclosed T-SR space, enclosing both RyR and Nav1.4 or 1.5 (Bardsley et al., 2021). The resulting elevated bulk [Ca^2+^]_i_ then initiates muscle contraction. It also exerts negative feedback actions on its initiating RyR-mediated Ca^2+^ release (Pagala and Taylor, 1998). SERCA1 or SERCA2 and plasma membrane Ca^2+^ transporters (PMCA) then return the released Ca^2+^ into the ER for sequestration by high concentrations of specialized low-affinity, high-capacity buffer molecules, such as calsequestrin, or to the extracellular space (Clapham, 2007). This restores the baseline low cytosolic [Ca^2+^]_i_. Surface membrane excitation is finally terminated by voltage-gated SkM Kv1 and/or cardiac muscle Kv4 K^+^ channel action.

Hippocampal pyramidal CA1 neurons similarly possess surface voltage-gated Na^+^, Ca^2+^, and K^+^, and intracellular Ca2^+^ release RyR channels. They also show endoplasmic reticular (ER)–plasma membrane (PM) junctions (EPJs) with clustered and functionally coupled L-type Ca^2+^ (LTCCs) and RyR channels. However, differing channel subtypes are involved. Of surface membrane ion channels, CA1 pyramidal neurons express Nav 1.1, 1.2, and 1.6 amongst Na^+^ channels (Wang et al., 2012), and Cav1.3, Cav2.3, and Cav3.x, activated by different magnitudes of membrane depolarization, amongst Ca^2+^ channels (Armstrong and Matteson, 1985). Among intracellular RyR-Ca^2+^ release channels, anti-RyR antibody labeling methods (Furuichi et al., 1994; Giannini et al., 1995) indicated expression of all three established RyR1-3 subtypes (McPherson and Campbell, 1993) in neuronal cells. More recent studies in RyR3^−/−^ mouse CA1 cells suggested important functional roles of Ca^2+^-induced Ca^2+^ release by highly expressed RyR3 in potentiating slow afterhyperpolarizing current, sIAHP (Tedoldi et al., 2020). In addition, Cav1.3–RyR3 interactions are promoted by Kv2.1 K^+^ channels present within the resulting Kv2.1–LTCC–RyR triads (Johnson et al., 2018; Vierra et al., 2019). Recently, in a mouse model expressing green fluorescent protein (GFP)-tagged RyR2, a specific GFP probe demonstrated high RyR2 expression in soma and dendrites, but not dendritic spines or presynaptic terminals, of CA1 pyramidal neurons or dentate gyrus granular neurons (Hiess et al., 2022).

Among K^+^ channels, the delayed rectifier Kv2.1, in addition to modifying neuronal excitability (Martina et al., 1998) to extents dependent on metabolic state (Misonou et al., 2005), enhances LTCC opening at polarized membrane potentials. Conversely, Cav3.1 and Cav1.2 channels were implicated in modulating Kv4.2 in hippocampal dendrites (Anderson et al., 2010; Murphy et al., 2022). Finally, their parallel increases and decreases in amplitude in intact neurons could be a basis for a dependence of transient outward *I*_K_ upon entry of extracellular Na^+^ (Marrero and Lemos, 2003). The latter findings parallel features of Na^+^-dependent K^+^ current activation in a different K^+^ channel subtype (Bhattacharjee and Kaczmarek, 2005; Hage and Salkoff, 2012). In this connection, the RyR antagonist dantrolene was recently proposed as a novel treatment for Alzheimer’s disease (Liang and Wei, 2015).

These similarities and differences in muscle and neuronal interactions between surface and intracellular membranes predict corresponding functional similarities and differences between them. Furthermore, native CA1 neurons in hippocampal slices may be amenable to study by similar pharmacological and biophysical methods as skeletal muscle. Ca^2+^ imaging methods had similarly demonstrated, respectively, enhanced and reduced Ca^2+^ transient amplitudes following challenge by low and high caffeine concentrations, and these effects were similarly occluded by the pharmacological depletion of intracellular Ca^2+^ stores (Sandler and Barbara, 1999). We here introduce loose-patch clamp methods (Stühmer et al., 1983) to investigate corresponding effects in native *in situ* CA1 pyramidal neurons in hippocampal coronal slices for the first time. This approach complements conventional tight patch electrode techniques previously used in both on- and whole-cell electrophysiological single-cell current-clamp (Liu et al., 2001; Minlebaev et al., 2013; Ghasemi et al., 2018; Vazetdinova et al., 2022) or voltage-clamp studies of neuronal cells in hippocampal tissue slices (Kodirov, 2023) or following isolation (Mathias et al., 1990; Klee et al., 1995). However, on the one hand, the whole-cell conventional patch clamp configuration permitting membrane current measurement over the entire somal cell membrane involves cell membrane rupture to electrically access the intracellular environment (French et al., 1990). The dialysis of often Ca^2+^-chelating ethylene glycol-bis (β-aminoethyl ether)-N, N, N′, N′-tetra acetic acid (EGTA)-containing pipette solution into the intracellular environment could then itself perturb intracellular Ca^2+^ dynamics (Leech & Holz IV, 1994; Perkins, 2006). Furthermore, neither this nor perforated patch variants permit multiple experimental seal formations and detachments with the same pipette before and following pharmacological challenges. Finally, in one report, it was not possible to control potential during the early sodium current transient, likely due to currents generated at a distance from the soma in the axon or dendrites (French et al., 1990). On the other hand, with the small pipette diameters, on-cell techniques leave intact membrane confine readings to relatively small localized membrane areas (Magee and Johnston, 1995; Perkins, 2006).

## 2 Materials and methods

### 2.1 Experimental strategy

All experimental procedures were approved by and conformed to the guidelines of the Animal Experiment Ethical Committee of the University of Surrey, Guildford, UK (NASPA-1819-25). All reagents were purchased from Sigma-Aldrich, Kent, UK, unless otherwise stated. The patches were acquired in the stratum pyramidalis (SP) layer of the CA1 hippocampus. Here, depolarization-activated currents were recorded before and following the administration of extracellularly bath-applied drugs. This approach allowed comparisons before and after drug application from each patch, thereby avoiding variations arising from maximum current amplitude differences between patches. Each experimental protocol was limited to one patch from any individual brain slice, thereby avoiding effects from prior drug exposure on other cells within that slice. Additionally, to maximize statistical independence, each experimental condition was only applied to a single hippocampal slice extracted from each brain.

### 2.2 Animals

Four-week-old C57BL/6 male mice (Charles River UK Ltd., Margate, United Kingdom) were housed under controlled conditions (ambient temperature 23°C ± 2°C, 12-h light/dark cycle) with food pellets and water supplied *ad libitum*. Animals were subjected to a 1-week adaptation period to animal house conditions prior to experiments. After this time, they were sacrificed by cervical dislocation (Schedule 1, UK Animals (Scientific Procedures) Act 1986).

### 2.3 Tissue preparation

Once sacrificed, the animals were rapidly decapitated, and the brain was harvested and placed in ice-cold HEPES-buffered holding artificial cerebrospinal fluid (aCSF) containing (in mM) 92 NaCl, 2.5 KCl, 30 NaHCO_3_, 1.25 NaH_2_PO_4_, 20 HEPES, 25 glucose, 10 MgCl_2_, and 0.5 CaCl_2_. The pH was adjusted to 7.4, and the solution was constantly bubbled with a mixture of 95% O_2_ and 5% CO_2_ (Ting et al., 2014). From these, 300-μm-thick coronal hippocampal slices were cut using a micro-slicer (7000smz-2 vibratome, Campden Instruments Ltd., UK). Slices were incubated for 1 h at room temperature (20°C–25°C) in HEPES-buffered holding aCSF, constantly bubbled with 95% O_2_ and 5% CO_2_. This minimized edema formation (MacGregor et al., 2003) and cell swelling of the neurons in the superficial layer of the tissue in contact with the blade during cutting procedures.

From each brain, four coronal slices were obtained from the anterior and medial hippocampal regions. From each slice, a single patch was selected for protocol application. Once obtained, the electrophysiological properties of the patch were tested by applying a depolarizing test pulse from the resting membrane potential (RMP) to (RMP + 80) mV. Only patches displaying clear-cut inward and outward currents were selected for further experimentation. Slices were preserved in HEPES-buffered holding aCSF and constantly bubbled with a mixture of 95% O_2_ and 5% CO_2_ for up to 5 h.

### 2.4 Bath setup and perfusion apparatus

A single coronal slice was placed in a bath chamber filled with 30 mL of standard recording aCSF containing (in mM) 124 NaCl, 2.5 KCl, 1.25 NaH_2_PO_4_, 24 NaHCO_3_, 5 HEPES, 12.5 glucose, 2 MgCl_2_, and 2 CaCl_2_ at a pH of 7.3–7.4 and a temperature T of 23°C–25°C. The bath was perfused through two peristaltic pumps for influx and efflux of solution (model 101UR, Watson-Marlow, Cornwall, UK), both set at a 4 mL/min flow rate to reduce disturbance to the tissue and acquired patch. The perfusing solutions were equilibrated for > 1 h at room temperature prior to experimentation, constantly perfused with fresh standard recording aCSF, and bubbled with a mixture of 95% O_2_ and 5% CO_2_.

### 2.5 Loose patch pipette manufacture and deployment

Patch pipettes were fabricated from borosilicate glass capillary tubes (GC150-10; Harvard Apparatus, Cambridge, UK) using a micropipette vertical puller (Model P-97 Sutter Instrument Co., Novato, CA) to obtain a progressive taper, at the end of which a square tip with a 20–25 µm diameter was achieved without fire polishing. The pipette was mounted into a 45° inclined pipette holder (model Q45W-B15P, Warner Instruments, Hamden, CT, USA), connected to a chloridized silver wire. This was inserted on the head stage of the loose-patch amplifier and held at 45° such that the pipette tip approached the slice surface with a perpendicular angle. In standard recording of aCSF, the average pipette resistance (R_pip_) recorded was ∼ 200 kΩ.

### 2.6 Loose patch clamp recording

**FIGURE 1:**
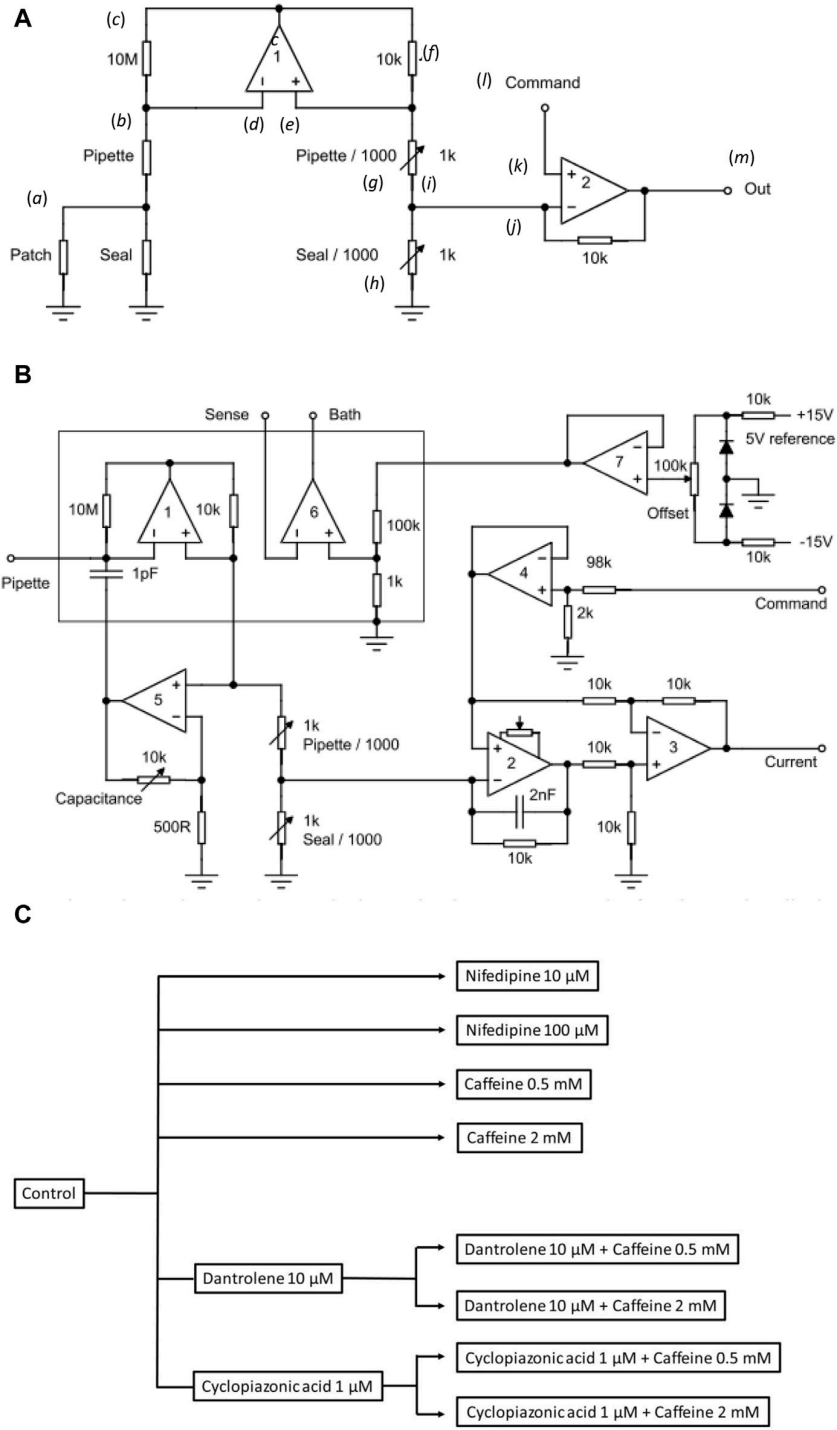
Experimental study design. **(A)** Basic layout illustrating the loose-patch clamp technique. **(B)** Outline of bespoke circuit components. **(C)** Pharmacological study design.

Figures 1A, B review the overall design of the particular custom-built loose-patch amplifier circuit that was used (Almers et al., 1983). It differs from conventional patch clamp recording circuitry in incorporating corrections for significantly larger seal leakage currents through a seal resistance R_seal_ <1 MΩ as opposed to many GΩ, which the pipette tip makes with the membrane. This additionally leads to larger series resistance R_pip_ errors through the pipette length. Both involve corrections so that the membrane patch was clamped to the command potential and the circuit output corresponded to the current flowing through the patch only. Nevertheless, this approach (Stühmer et al., 1983) has been previously adapted and validated in electrophysiological studies of amphibian (Chin et al., 2004), mammalian skeletal (Matthews et al., 2019; Sarbjit-Singh et al., 2020; Liu et al., 2021), and cardiac atrial (Valli et al., 2018a) and ventricular muscles (Ahmad et al., 2019).

In Figure 1A, the junction between the pipette resistance (R_pip_) and seal resistance (R_seal_) (*a*) is to be ‘clamped’. The back of the pipette (*b*) connects to both the 10 MΩ resistor (*c*) and inverting input of op amp 1 (*d*). The non-inverting input of op amp 1 (*e*) connects to a 10 KΩ resistor (*f*). Op amp 1 then adjusts its output voltage to minimize the voltage difference between its two inputs. The right-hand output of op amp 1 mirrors point (*c*) with the 10K resistor (*f*), and resistors pipette/1,000 of resistance R_pip_/1,000 (*g*), and seal/1,000 of resistance R_seal_/1,000 (*h*), each accordingly of resistance 1/1,000 those of the corresponding elements on the opposite side. The current flowing through the 10 kΩ resistor is then 1,000 times greater than that from the loose-patch pipette flowing through the 10 MΩ resistor. The junction (*i*) between pipette/1,000 and seal/1,000 then connects to the inverting terminal of clamping op amp 2 (*j*). The latter is then compared to the input to its non-inverting terminal (*k*) from the command voltage step (*l*). The output voltage of Op amp 2 is adjusted to minimize the voltage difference between its two inputs. This corresponds to the current required to voltage-clamp the junction between the pipette/1,000, and seal/1,000 (*m*) and therefore the junction between the pipette and seal resistors. This circuitry, thus, corrects for much greater leaks and consequently pipette currents. Here, the true membrane current is much smaller than the leak current and the latter must be corrected out before feeding into the actual patch clamp circuit. Delivery of such greater currents is potentially limited by the ability of the Ag/AgCl junction for sustained current delivery through the loose-patch electrode.

The values of the different resistors require optimizing for any given application and preparation against the different electrode tip diameters, leak currents, and pipette resistances (Yan et al., 2020). The latter and details of the clamping configuration are summarized in more detail in Figure 1B. Here, in addition to op amps 1 and 2 in Figure 1A, op amp 3 subtracts the command potential offset from the output of Op amp 2. The final output voltage, thus, represents the patch current. Op amp 4 buffers the command input. Op amp 5 presents a scaled replica of the pipette voltage to a capacitor connected to the pipette input; the scale factor is adjustable to cancel pipette capacity currents. Op amp 6 voltage clamps the bath to a voltage determined using the offset potentiometer. Op amp 7 cancels junction potentials to ensure zero current flow when the pipette is clamped to nominally zero voltage.

Prior to patch clamp measurements, manual calibrations and balancing set the values of the pipette/1,000 and seal/1,000 resistors. In the bath mode, the seal/1,000 resistor is shorted out. This enables pipette resistance measurement with the application of square-wave voltage clamp pulses. Pipette/1,000 is then adjusted to cancel the square-wave leakage current through the pipette resistance R_pip_. In the patch mode, the resistors are reconnected. The contact between the pipette and membrane generates a change in the magnitude of the uncompensated currents evoked by small-amplitude voltage clamp pulses, reflecting an increase in resistance at the pipette tip. The seal is then stabilized through the application of negative pressure through the electrode. On such patch seal formation, the leak/1,000 control is adjusted to cancel the seal current and compensate for R_seal_. Thus, in the operation of the circuit, the variable resistances of the compensating bridge circuit are adjusted to match the voltage drops across both R_pip_ and R_seal_. Accordingly, the membrane patch is clamped to the command potential, and the circuit output corresponds to the current flowing through the patch only. In standard recording aCSF, the average R_pip_ recorded was ∼ 180 kΩ. Average R_seal_ varied between patches and ranged between 1.5 and 2.0 times the value of R_pip_ (average ∼ 300 kΩ). The patch was then examined for the presence of membrane currents through a 25 ms depolarizing step to (RMP + 80). Viable patches displaying clear-cut inward and outward currents were tested with clamp steps over a range of depolarizing voltages to obtain a family of current responses.

### 2.7 Recording protocol

Voltage clamp steps were delivered using an IBM-compatible computer. As the pipette clamped the extracellular face of the patch, the applied voltage steps produced membrane potential excursions of opposite sign to the conventionally expressed membrane potential and were relative to the resting membrane potential (RMP); they are accordingly referred to as such in this report. Each recording had a duration of 30 ms. At the beginning of each clamp step protocol, the patch was maintained at RMP (holding potential) for 1 ms. Each protocol used a 5 ms hyperpolarizing pre-pulse at (RMP—40) mV to relieve any residual Nav inactivation at the RMP. This was followed by a test pulse of variable amplitude and 15 ms duration, starting at (RMP—40) mV and altered in (RMP + 10) mV increments until a maximum test voltage of (RMP + 120) mV was reached. The RMP was finally restored at the end of the 21 ms clamp step protocol, and the currents were recorded for a further 9 ms. Any remaining uncompensated leak current was adjusted using a P/4 procedure, which involved delivering four voltage clamp steps of opposite polarity and a quarter of the magnitude of the test pulse immediately after it. As the P/4 pulses covered voltage ranges that would not activate any voltage-gated conductance, they solely represented leak currents. These leak currents were eliminated by adding them to the recorded test pulse current. A series of membrane-depolarizing clamp steps was used to derive current–voltage curves reflecting channel activation in conjunction with the P/4 pulse procedure.

Data were sampled at a 50 kHz digital sampling rate and filtered over a DC-10 kHz bandwidth using a 10 kHz Bessel low-pass filter. The region corresponding to the SP layer of the hippocampal CA1 was optically identified. All the experiments were conducted at room temperature (20°C–25 °C). The data were digitized and stored using custom-made loose-patch clamp software.

### 2.8 Administration of drugs

After seal acquisition, the bath was perfused with a succession of recording aCSF solutions with the aid of perfusion pumps while monitoring R_seal_. Perfusion was maintained at a steady flow rate throughout to both ensure seal preservation and maintain tissue viability. A first family of control currents was obtained in standard recording aCSF in the presence or absence of conditioning reagents. The relevant, standardized pulse procedure took <10 min. This was followed by the replacement of perfusate with fresh standard test aCSF containing test drugs at the defined concentrations. The total volume of the washout replacement solution (120 mL) exceeded four times that of the bath. Each such solution change accordingly took a period of <30 min. This ensured complete bath solution replacement and avoided diluting effects between the two solutions. The next family of test currents was then obtained using the same voltage clamp protocol. The overall duration of the entire perfusate replacement/recording protocol accordingly took <1 h. This protocol, using a constant flow of perfusing solution, permitted all recordings to be obtained from the same undisrupted patch.

The tested compounds were nifedipine at 10 and 100 μM, caffeine at 0.5 and 2 mM, dantrolene at 10 μM, and CPA (Bio-Techne Ltd, UK) at 1 μM (Figure 1C). Nifedipine at 10 and 100 μM was used to assess the impact of Cav blockage on CA1 pyramidal neuron membrane current. Caffeine was used at low (0.5 mM) RyR agonist and high (2 mM) inhibitory concentrations. Dantrolene (10 μM) and CPA (1 μM) were used, respectively, as RyR and SERCA blockers. The combined effects of caffeine and dantrolene/CPA were tested by pre-incubating hippocampal slices with either 10 μM dantrolene or 1 μM CPA, followed by additional inclusion of different caffeine concentrations. In common with the limited specificity shown by most pharmacological agents, these agents individually could exert respective effects in addition to those on RyR-mediated Ca^2+^ release or SERCA-mediated Ca^2+^ re-uptake. The former is exemplified by caffeine-mediated phosphodiesterase, in addition to RyR inhibition. However, the present findings from their use in combination likely concern their common actions directed at cellular Ca^2+^ homeostasis.

### 2.9 Statistical analysis

Current–voltage plots, where currents were normalized to the patch area as inferred from the pipette diameter and presented as current densities (means ± SEM), were derived from the recordings. Current means recorded in pre- and post-treatment conditions, *I* (pA/µm^2^), were statistically analyzed through a within-patch paired *t*-test using GraphPad Prism^®^ software version 6 for Windows (*p* < 0.05). Multiple groups of data were tested using a one-way ANOVA plus a *post hoc* Tukey test. Sample sizes are described as N_1_ for the number of brains and n for the number of patches. All statistics are based on n.

## 3 Results

### 3.1 Extracellular Na^+^ replacement removes inward and transient outward currents

The loose-patch recordings in the CA1 pyramidal neurons demonstrated transient inward, followed by transient outward, and sustained outward currents in response to families of depolarizing steps. This agreed with previous findings from the technique in other neuronal cell types that had implicated Na^+^ (*I*_Na_) and K^+^ currents (*I*_K_) in these deflections (Marrero and Lemos, 2003). Initial control experiments investigating the effects of extracellular ionic substitutions confirmed these earlier reports. They investigated the effects of NaCl replacement first by N-methyl-d-glucamine (NMDG) chloride and then, in separate experiments, by choline-Cl.

**FIGURE 2:**
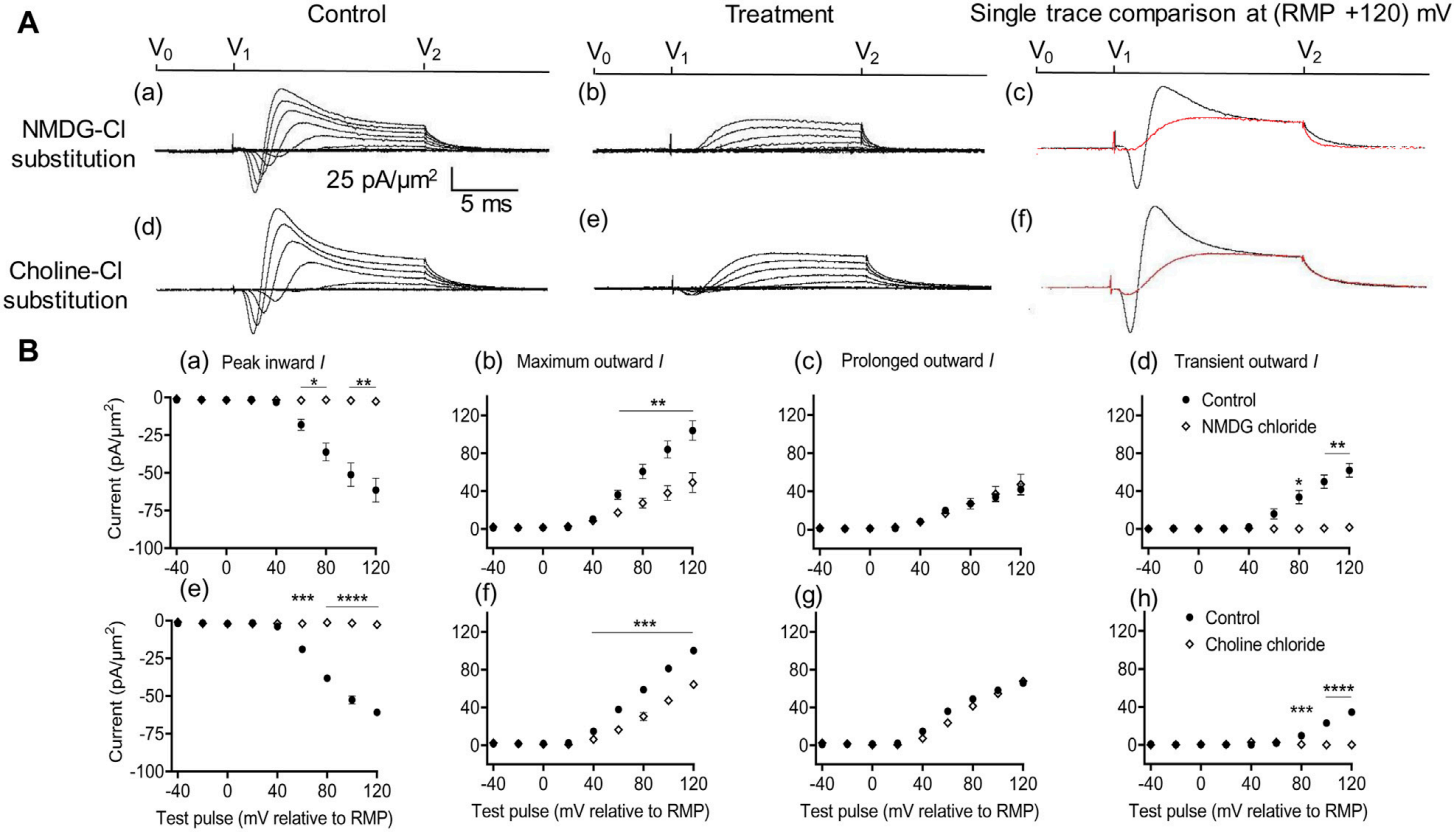
Extracellular Na^+^ replacement blocks inward and reduces transient outward currents. **(A)** Effect of NaCl replacement by N-methyl-d-glucamine (NMDG) (top row) (R_seal_: 510 kΩ, pipette diameter: 24 μm) and choline chloride (bottom row) (R_seal_: 285 kΩ, pipette diameter: 24 μm). From left to right: typical results before and following treatment and superimposed comparisons of single traces obtained at (RMP + 120) mV. Control currents recorded under standard recording aCSF containing (in mM): 124 NaCl, 2.5 KCl, 1.25 NaH_2_PO_4_, 24 NaHCO_3_, 5 HEPES, 12.5 glucose, 2 MgCl_2_, and 2 CaCl_2_ at a pH of 7.3–7.4 and T = 23°C–25°C. In choline-Cl experiments, the sole NaCl was replaced by the same molarity of choline chloride. For NMDG experiments, the solution used contained (in mM) 140 NMDG-Cl, 3 KCl, 10 HEPES, 25 glucose, 2 MgCl_2_, and 2 CaCl_2_ at a pH of 7.3–7.4 and T = 23°C–25°C.**(B)** Mean current densities normalized to the pipette diameter for peak inward, maximum outward, prolonged outward, and transient outward currents for NMDG-Cl (top row: **(a–d)**), respectively and choline-Cl substitution experiments (bottom row: **(e–h)**), respectively (average R_seal_: 420 kΩ). RMP, resting membrane potential. N_1_ = 4 and n = 4 for NMDG-Cl substitution. N_1_ = 5 and n = 5 for choline-Cl substitution. Significance levels between pre- and post-treatment groups. *p < 0.05, **p < 0.01, ***p < 0.001, and ****p < 0.0001.

The experiments first obtained current families in response to depolarizing steps applied in membrane patches in slices perfused with standard artificial cerebrospinal fluid (aCSF) (Figure 2A). The pipette was then lifted, and the extracellular solution replaced by a Na^+^-free perfusate in which NaCl was replaced with isomolar NMDG-Cl or choline-Cl. The seal was then re-established by lowering the pipette using the same micromanipulator coordinates, and a new family of currents was recorded. In addition to obtaining current families and displaying current density normalized to the pipette diameter, their mean currents were plotted against voltage excursion. Prolonged outward currents were quantified from current amplitudes at the end of the voltage steps, and transient outward current components were estimated from the difference between the latter and the maximum outward current amplitudes (Figure 2B).

With both substitutions, Na^+^ replacement both blocked peak inward and reduced transient outward but did not significantly affect prolonged outward current. This was reflected in mean (±SEM) current amplitudes at (RMP+120) mV in the presence of NMDG for inward (61.49 ± 7.94 pA/μm^2^ pre-administration vs. 2.74 ± 0.11 pA/μm^2^ post administration, *p* = 0.0053, n = 4), maximum (104.1 ± 10.31 vs. 49.07 ± 10.4 pA/μm^2^, *p* = 0.0072), transient (62.08 ± 7.25 vs. 1.84 ± 0.38 pA/μm^2^, *p* = 0.0037), and prolonged outward currents (42 ± 5.83 vs. 47.23 ± 10.65 pA/μm^2^, *p* = 0.6537). Corresponding values in the presence of choline were inward (60.9 ± 1.66 pA/μm^2^ pre administration vs. 2.74 ± 0.08 pA/μm^2^ post administration, *p* < 0.0001, n = 5), maximum (100.24 ± 0.17 vs. 64.27 ± 2.65 pA/μm^2^, *p* = 0.0002), transient (34.49 ± 0.22 vs. 0 ± 0 pA/μm^2^, *p* < 0.0001), or prolonged outward currents (65.74 ± 0.4 vs. 67.85 ± 0.5 pA/μm^2^, *p* = 0.0705). This agreed with the previous results, which accordingly identified the inward with an *I*_Na_ and with previous reports of a possible Na^+^ dependence of the outward transient *I*_K_ (Marrero and Lemos, 2003). The latter has been previously associated with outward Na^+^-activated K^+^ channels (Slack and Slick, KCNT1 and KCNT2 channels), known to produce delayed outward currents (Bhattacharjee and Kaczmarek, 2005; Hage and Salkoff, 2012).

### 3.2 No significant actions of nifedipine on inward and outward membrane currents

**FIGURE 3:**
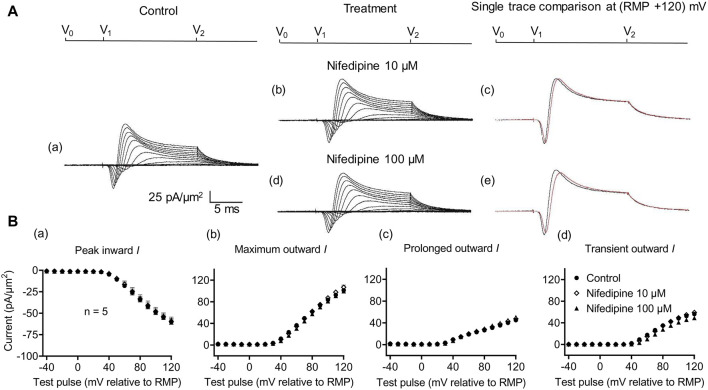
Activation properties of inward and outward currents of murine CA1 pyramidal neurons under loose-patch clamp in response to treatment with the membrane Ca^2+^ channel blocker nifedipine. **(A)** Currents elicited by progressively depolarizing voltage steps are shown for a typical patch (R_seal_: 260 kΩ and pipette diameter: 22 μm) under pre-treatment control conditions **(a)** and following treatment with 10 μM **(b)** and 100 μM **(d)** nifedipine. Subpanels **(c)** and **(e)** display single-trace comparisons between control and treatment at the most depolarized voltage tested, (RMP + 120) mV, for 10 μM **(c)** and 100 μM nifedipine **(e)**. **(B)** Mean (±SEM) currents plotted against voltage excursion: the resulting current–voltage curves, expressing current density normalized to the pipette diameter shown for control (filled circles), 10 μM nifedipine (empty rhombuses), and 100 μM nifedipine (filled triangles) peak inward **(a)**, maximum outward **(b)**, prolonged outward **(c)**, and transient outward currents **(d)**. Average R_seal_: 300 kΩ. RMP, resting membrane potential. N_1_ = 5 and n = 5.

Further controls excluded Ca^2+^ current contributions to, or their effects on, inward currents. These explored effects of low (10 μM) and high nifedipine concentrations (100 μM) known, respectively, to block selectively LTCCs and remaining Cav types (Curtis and Scholfield, 2001) (Figure 3). Neither concentration affected the inward currents (peak values at the largest depolarizations: controls: 60.37 ± 2.70 pA/μm^2^; following the addition of nifedipine at 10 μM: 59.46 ± 3.23 pA/μm^2^, *p* = 0.9745, n = 5; at 100 μM: 57.4 ± 2.9 pA/μm^2^ post-administration, *p* = 0.7620, n = 5 in each group). Maximum, transient, and prolonged outward currents were all similarly unaffected (maximum outward current: control: 101.22 ± 3.33 pA/μm^2^, nifedipine, 10 μM: 106.83 ± 4.13 pA/μm^2^, *p* = 0.455; 100 μM: 99.52 ± 1.63 pA/μm^2^, *p* = 0.925. Transient outward current: control: 56.23 ± 2.47 pA/μm^2^, nifedipine, 10 μM: 59.25 ± 3.43 pA/μm^2^, *p* = 0.7843; 100 μM: 48.94 ± 3.51 pA/μm^2^, *p* = 0.2736. Prolonged outward current: controls: 45 ± 3.13 pA/μm^2^; nifedipine, 10 μM: 47.59 ± 3.42 pA/μm^2^, *p* = 0.8441; 100 μM: 48.58 ± 3.29 pA/μm^2^, *p* = 0.4730). These findings exclude significant surface membrane Ca^2+^ current contributions to the observed inward currents observed here. They also exclude the participation of such Ca^2+^ currents in the inward current alterations that followed the experimental manipulations described below.

### 3.3 Agonist (0.5 mM) but not antagonist (2 mM) caffeine concentrations inhibit inward and potassium current

**FIGURE 4:**
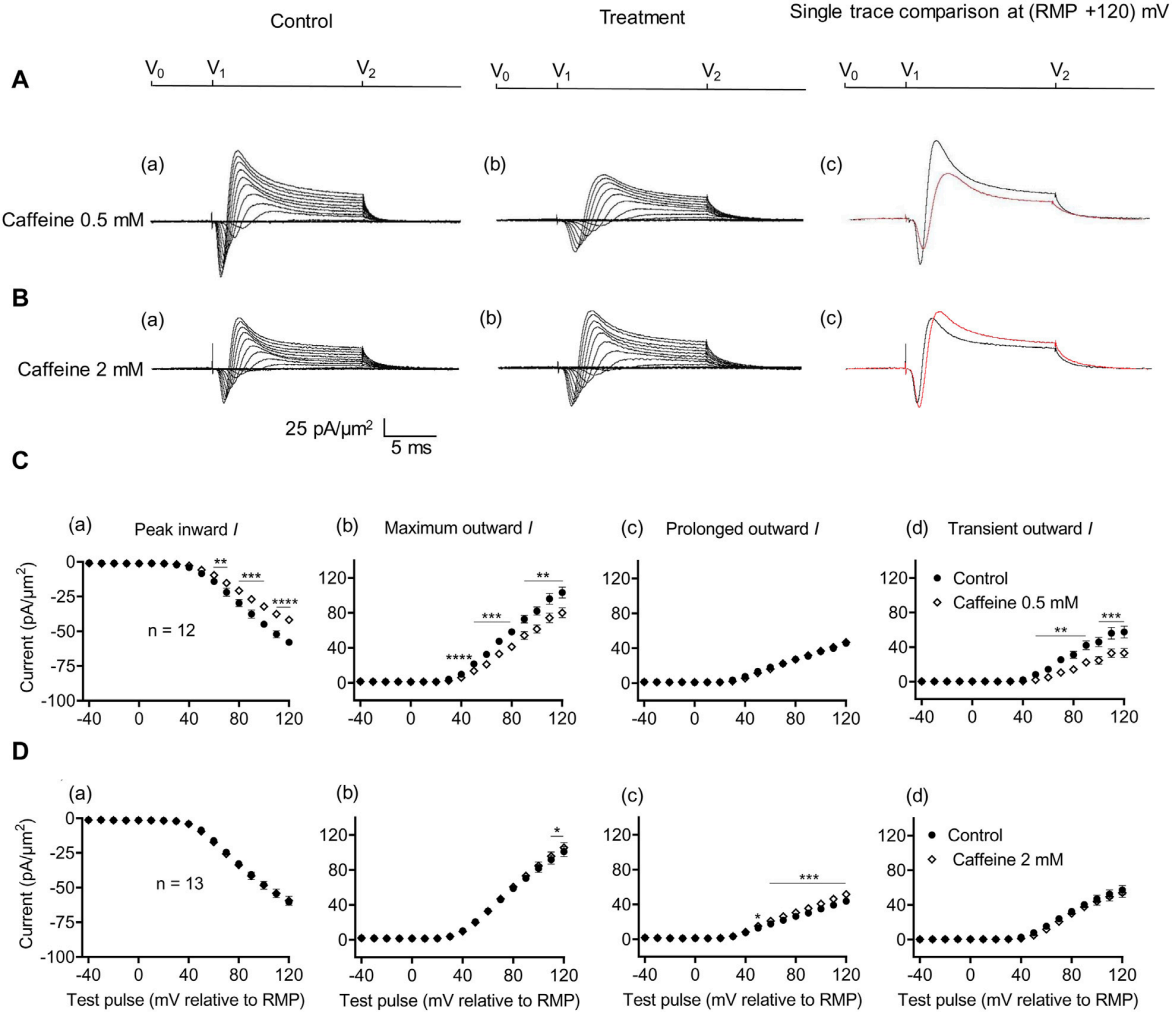
Activation properties of inward and outward currents of murine CA1 pyramidal neurons under loose-patch clamp in response to treatment with low (0.5 mM) and high (2 mM) caffeine concentrations. **(A, B)** Currents elicited by progressively depolarizing voltage steps are shown for an example patch (R_seal_: 260 kΩ; pipette diameter: 22 μm) under pre-treatment control conditions **(a)** and following treatment **(b)** with 0.5 mM **(A)** and 2 mM caffeine **(B)**. Subpanels **(c)** display single-trace comparisons between control and treatment at the most depolarized voltage tested, (RMP + 120) mV. RMP, resting membrane potential. **(C, D)** Current–voltage curves for current components before and following caffeine challenge. Mean (±SEM) currents plotted against voltage excursion: the resulting current–voltage curves, expressing current density normalized to the pipette diameter are shown for control currents and treatment with 0.5 mM **(C)** and 2 mM caffeine **(D)** for peak inward **(a)**, maximum outward **(b)**, prolonged outward **(c)**, and transient outward currents **(d)**. Data points from control currents are displayed as filled circles, while data points acquired after treatment administration are displayed as empty rhombuses. Average R_seal_: 300 kΩ. RMP, resting membrane potential. N_1_ = 5 and n = 5.

The effects of RyR-mediated modulations of [Ca^2+^]_i_ on CA1 pyramidal neuron membrane currents before and following applications of the RyR agonist caffeine at low activating (0.5 mM) and high inactivating concentrations (2 mM) were compared in separate experiments. Caffeine (0.5 mM) decreased maximum amplitudes of peak inward current (57.94 ± 2.17 pA/μm^2^ pre-administration vs. 41.82 ± 2.13 pA/μm^2^ post-administration, *p* < 0.0001, n = 12) (Figure 4A). This result agrees with its reported action of decreasing inward current in SkM (Sarbjit-Singh et al., 2020). It also decreased the maximum amplitude of the outward current (103.31 ± 6.37 vs. 80.04 ± 5.66 pA/μm^2^, *p* = 0.0047) consistent with effects on a Na^+^ dependent transient outward current (57.49 ± 6.85 vs. 33.11 ± 5.33 pA/μm^2^, *p* = 0.0007). It did not alter the prolonged outward current (45.82 ± 2.89 vs. 46.92 ± 2.64 pA/μm^2^, *p* = 0.6650) (Figure 4C). In contrast, caffeine (2 mM) affected neither inward (60.43 ± 2.42 vs. 59.48 ± 3.29 pA/μm^2^, *p* = 0.7841, n = 13) nor transient outward current (57.14 ± 5.15 vs. 53.94 ± 5.13 pA/μm^2^, *p* = 0.1719) (Figures 4B, D). This result differs from its reported actions increasing inward current in SkM (Sarbjit-Singh et al., 2020). There was a small increase in maximum outward current (101.00 ± 5.80 vs. 105.54 ± 5.76 pA/μm^2^, *p* = 0.0366). However, this may reflect increased prolonged current amplitudes (43.86 ± 3.43 vs. 52 ± 3.57 pA/μm^2^, *p* = 0.0002).

### 3.4 Effects of pre-incubation with the RyR antagonist dantrolene

**FIGURE 5:**
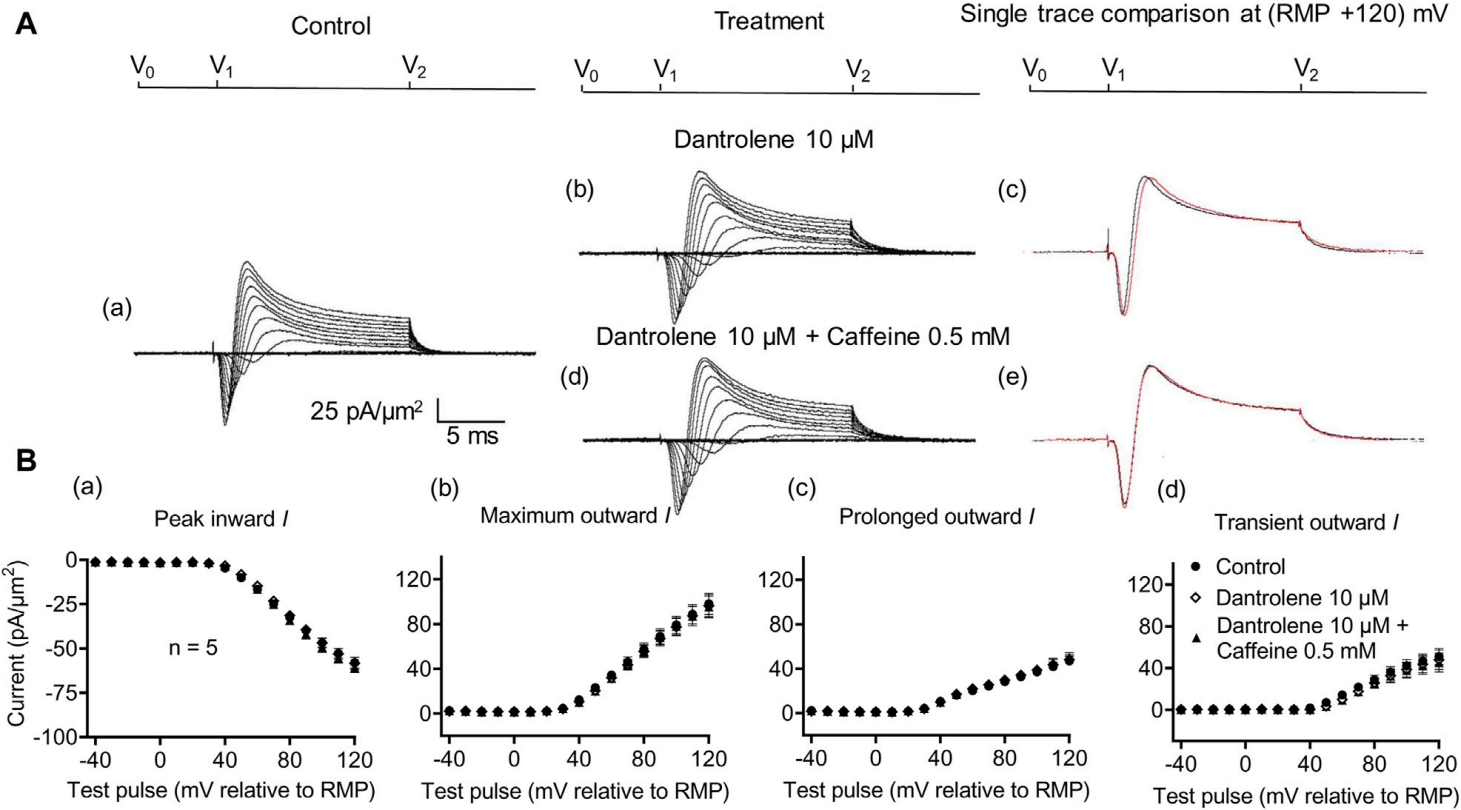
Activation properties of inward and outward currents of murine CA1 pyramidal neurons under loose-patch clamp in response to treatment with dantrolene and 0.5 mM caffeine. **(A)** Currents elicited by progressively depolarizing voltage steps are shown for a typical patch (R_seal_: 260 kΩ, pipette diameter: 22 μm) under pre-treatment control conditions **(a)** and following treatment with 10 μM dantrolene **(b)** and 10 μM dantrolene + 0.5 mM caffeine **(d)**. Subpanels **(c, e)** display single-trace comparisons between control and treatment at the most depolarized voltage tested, (RMP + 120) mV, for 10 μM dantrolene **(c)** and 10 μM dantrolene + 0.5 mM caffeine **(e)**. **(B)** Mean (±SEM) currents plotted against voltage excursion: the resulting current–voltage curves, expressing current density normalized to the pipette diameter, are shown for control (filled circles), 10 μM dantrolene (empty rhombuses), and 10 μM dantrolene + 0.5 mM caffeine (filled triangles) for peak inward **(a)**, maximum outward **(b)**, prolonged outward **(c)**, and transient outward currents **(d)**. Average R_seal_: 300 kΩ. RMP, resting membrane potential. N_1_ = 5 and n = 5.

**FIGURE 6:**
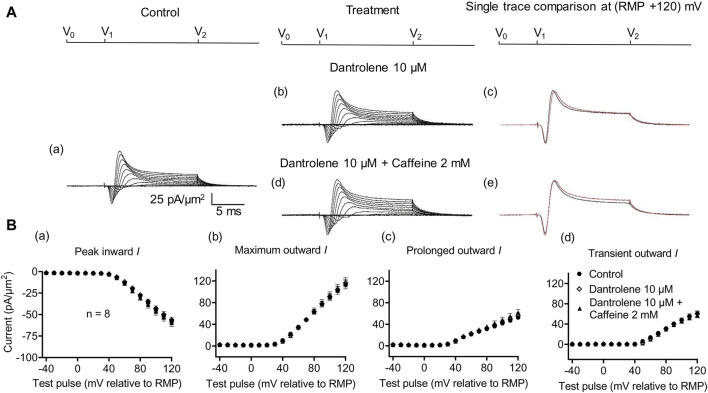
Activation properties of inward and outward currents of murine CA1 pyramidal neurons under loose-patch clamp in response to treatment with dantrolene and 2 mM caffeine. **(A)** Currents elicited by progressively depolarizing voltage steps are shown for typical patch (R_seal_: 260 kΩ; pipette diameter: 22 μm) under pre-treatment control conditions **(a)** and following treatment with 10 μM dantrolene **(b)** and 10 μM dantrolene + 2 mM caffeine **(d)**. Subpanels **(c, e)** display single-trace comparisons between control and treatment at the most depolarized voltage tested, (RMP + 120) mV, for 10 μM dantrolene **(c)** and 10 μM dantrolene + 2 mM caffeine **(e)**. **(B)** Mean (±SEM) currents plotted against voltage excursion: the resulting current–voltage curves, expressing current density normalized to the pipette diameter, are shown for control (filled circles), 10 μM dantrolene (empty rhombuses), and 10 μM dantrolene + 2 mM caffeine (filled triangles) for peak inward **(a)**, maximum outward **(b)**, prolonged outward **(c)**, and transient outward currents **(d)**. Average R_seal_: 300 kΩ. RMP, resting membrane potential. N1 = 5 and n = 5.

Dantrolene is known to block RyR activity selectively by decreasing the Ca^2+^ affinities of its activation sites, thereby stabilizing its closed states (Zhao et al., 2001). It also antagonizes caffeine action on RyR (Zhao et al., 2001). In SkM, dantrolene (10 μM) pre-administration by itself reduced background levels of *I*_Na_ inhibition, suggesting the effects of a reduced [Ca^2+^] within a possible local T-SR triadic domain (Bardsley et al., 2021). It then abrogated the effects on *I*_Na_ of subsequently added 0.5- or 2-mM caffeine (Sarbjit-Singh et al., 2020). Experiments extending these explorations to CA1 pyramidal neurons involved pre-incubating hippocampal slices for ∼ 10 min in 10 μM dantrolene, followed by replacement with an aCSF perfusate containing either 10 μM dantrolene + 0.5 mM caffeine or 10 μM dantrolene + 2 mM caffeine (Figures 5, 6).

Dantrolene pre-incubation in CA1 pyramidal neurons produced effects that *differed* from its previously reported SkM actions. Thus, it did not affect inward (57.89 ± 3 pA/μm^2^ pre-administration vs. 58.38 ± 3.32 pA/μm^2^ post-administration, *p* = 0.9920, n = 5), maximum (98.04 ± 9.54 vs. 96.15 ± 9.91 pA/μm^2^, *p* = 0.9897), transient (51.1 ± 7.61 vs. 47.92 ± 9.39 pA/μm^2^, *p* = 0.9642), or prolonged outward currents (46.94 ± 4.48 vs. 48.24 ± 4.77 pA/μm^2^, *p* = 0.9784) (Figure 5). However, in agreement with its SkM actions, it abrogated the actions of the additional inclusion of 0.5 mM caffeine (Figure 6). Caffeine now had no significant effects on inward (58.38 ± 3.32 vs. 60.77 ± 2.73 pA/μm^2^, *p* = 0.7827, n = 5), maximum (96.15 ± 9.91 vs. 95.33 ± 9.64 pA/μm^2^, *p* = 0.998), transient outward (51.1 ± 7.61 vs. 47.92 ± 9.39 pA/μm^2^, *p* = 0.8846), and prolonged outward currents (48.24 ± 4.77 vs. 50.09 ± 4.58 pA/μm^2^, *p* = 0.8808). The use of 2 mM caffeine similarly left inward (56.31 ± 3.27 vs. 59.74 ± 4.32 pA/μm^2^, *p* = 0.9191, n = 8), maximum (116.16 ± 7.23 vs. 118.47 ± 7.68 pA/μm^2^, *p* = 0.9754), transient outward (60.22 ± 4.72 vs. 56.73 ± 4.56 pA/μm^2^, *p* = 0.8219), and prolonged outward currents (55.94 ± 5.27 vs. 61.74 ± 5.93 pA/μm^2^, *p* = 0.4900) unchanged relative to results during dantrolene pre-treatment.

### 3.5 Cyclopiazonic acid abrogates the effects of 0.5 but not 2 mM caffeine

A complementary maneuver to the dantrolene challenge employed SERCA blockers such as thapsigargin or cyclopiazonic acid (CPA). These would ultimately impair ER Ca^2+^ store replenishment and, in turn, the background efflux of store Ca^2+^ into the cytosol (Seidler et al., 1989). In the previous report, in common with previously reported dantrolene actions (Sarbjit-Singh et al., 2020), CPA pre-administration both reduced background *I*_Na_ inhibition and abrogated the effects of subsequent caffeine challenges on skeletal muscle *I*_Na_ (Liu et al., 2021).

**FIGURE 7:**
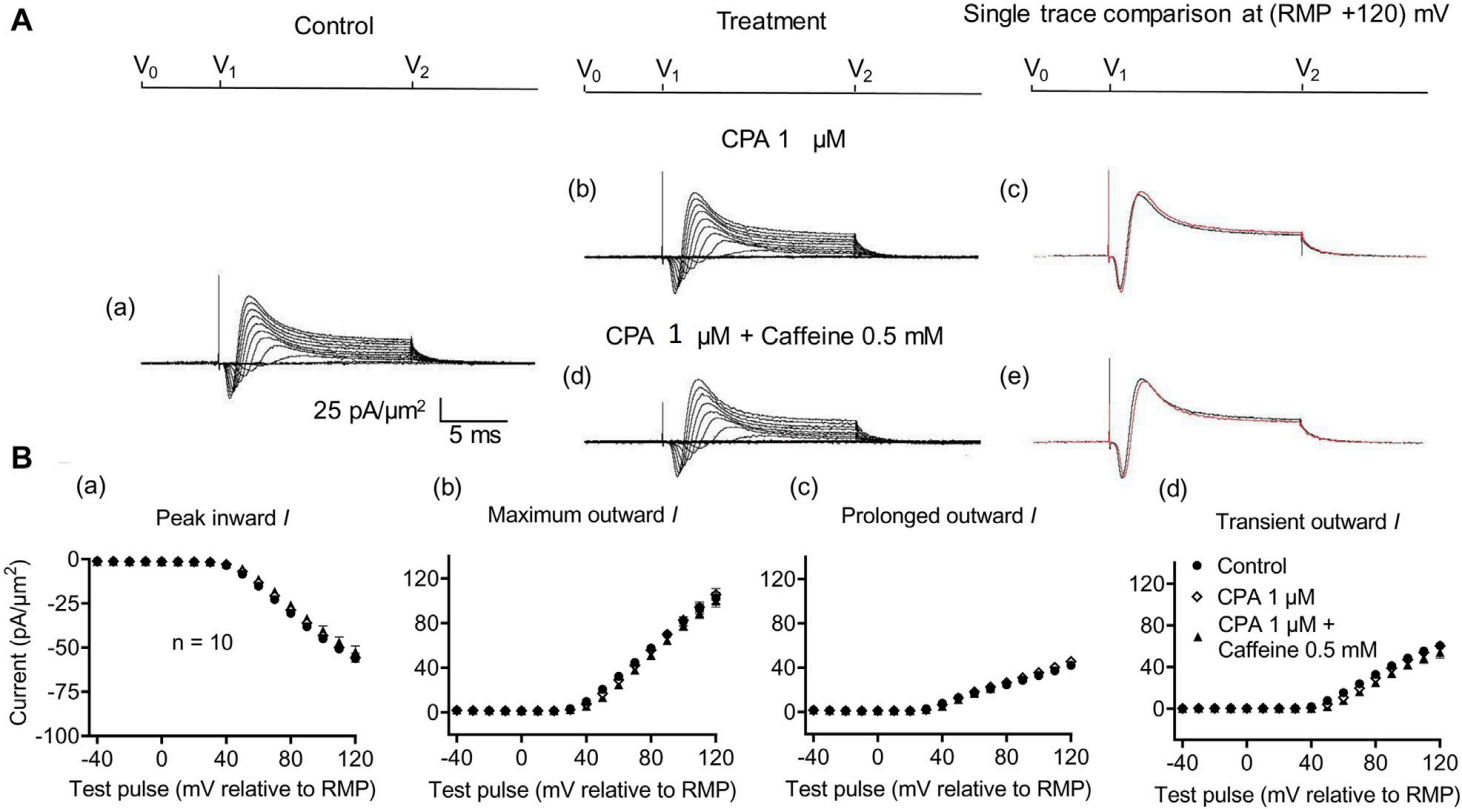
Activation properties of inward and outward currents of murine CA1 pyramidal neurons under loose-patch clamp in response to treatment with cyclopiazonic acid and 0.5 mM caffeine. **(A)** Currents elicited by progressively depolarizing voltage steps are shown for typical patch (R_seal_: 260 kΩ; pipette diameter: 22 μm) under pre-treatment control conditions **(a)** and following treatment with 1 μM CPA **(b)** and 1 μM CPA + 0.5 mM caffeine **(d)**. Subpanels **(c, e)** display single-trace comparisons between control and treatment at the most depolarized voltage tested, (RMP + 120) mV, for 1 μM CPA **(c)** and 1 μM CPA + 0.5 mM caffeine **(e)**. **(B)** Mean (±SEM) currents plotted against voltage excursion: the resulting current–voltage curves, expressing current density normalized to the pipette diameter, are shown for control (filled circles), 1 μM CPA (empty rhombuses), and 1 μM CPA + 0.5 mM caffeine (filled triangles). Current voltage curves shown for peak inward **(a)**, maximum outward **(b)**, prolonged outward **(c)**, and transient outward currents **(d)**. Average R_seal_: 300 kΩ. RMP, resting membrane potential. N_1_ = 5 and n = 5.

**FIGURE 8:**
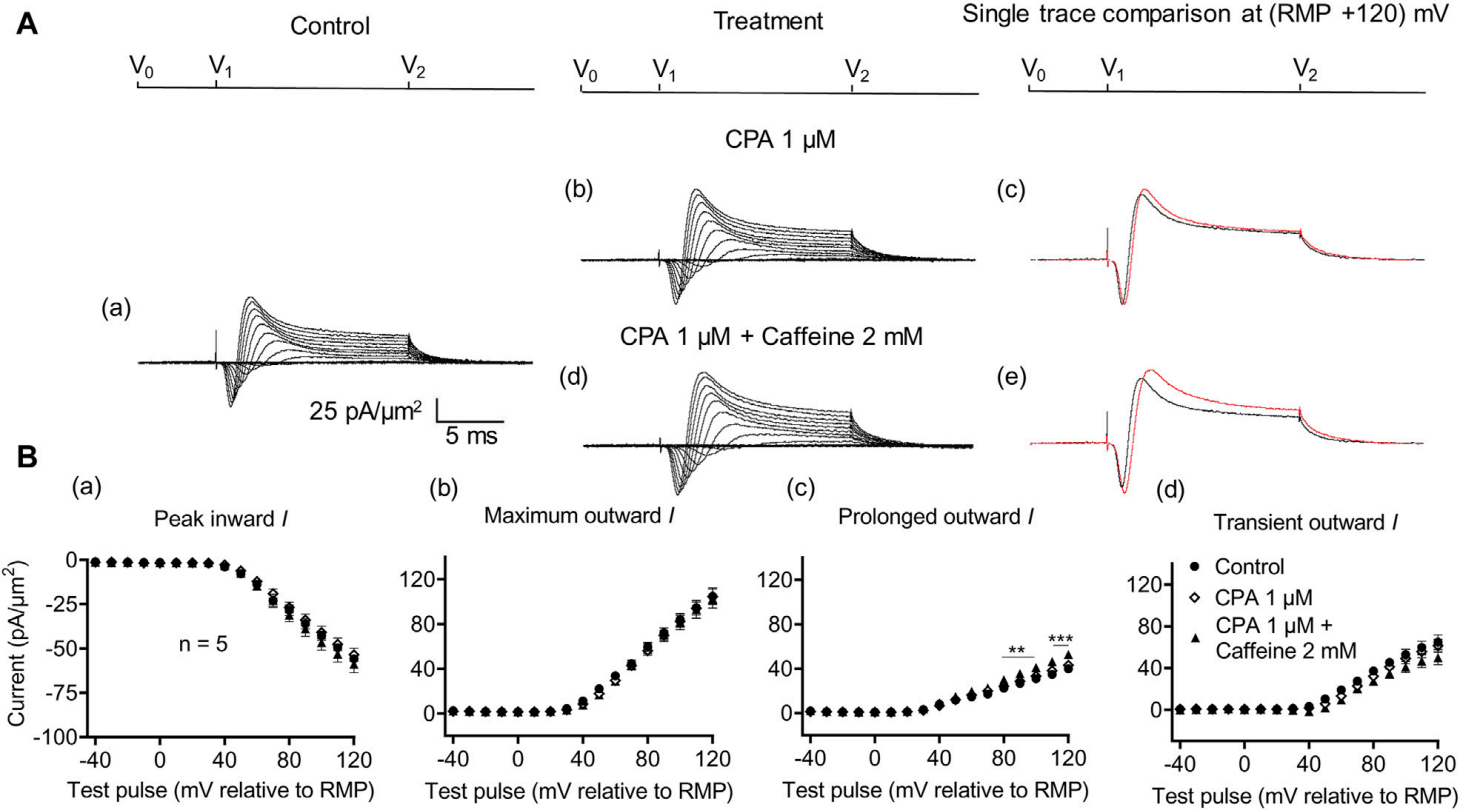
Activation properties of inward and outward currents of murine CA1 pyramidal neurons under loose-patch clamp in response to treatment with cyclopiazonic acid and 2 mM caffeine. **(A)** Currents elicited by progressively depolarizing voltage steps are shown for a typical patch (R_seal_: 260 kΩ; pipette diameter: 22 μm) under pre-treatment control conditions **(a)** and following treatment with 1 μM CPA **(b)** and 1 μM CPA + 2 mM caffeine **(d)**. Subpanels **(c, e)** display single-trace comparisons between control and treatment at the most depolarized voltage tested, (RMP + 120) mV, for 1 μM CPA **(c)** and 1 μM CPA + 2 mM caffeine **(e)**. **(B)** Mean (±SEM) currents plotted against voltage excursion: the resulting current–voltage curves, expressing current density normalized to the pipette diameter, are shown for control (filled circles), 1 μM CPA (empty rhombuses), and 1 μM CPA + 2 mM caffeine (filled triangles) for peak inward **(a)**, maximum outward **(b)**, prolonged outward **(c)**, and transient outward currents **(d)**. Average R_seal_: 300 kΩ. RMP, resting membrane potential. N_1_ = 5 and n = 5.

In the present experiments on CA1 pyramidal neurons, the initial 1 μM CPA administration did not affect inward current (56.2 ± 2.28 pA/μm^2^ pre-administration vs. 57.27 ± 2.8 pA/μm^2^ post-administration, *p* = 0.9931, n = 10) in contrast to its action in SkM (Liu et al., 2021) (Figure 7). It also left unchanged maximum (102.51 ± 5.04 vs. 105.61 ± 5.23 pA/μm^2^, *p* = 0.9074), transient outward (60.45 ± 3.65 vs. 60.11 ± 3.87 pA/μm^2^, *p* = 0.9984), and prolonged currents (42.05 ± 2.61 vs. 45.5 ± 2.94 pA/μm^2^, *p* = 0.6403). As with the case of dantrolene, the CPA pre-administration, abrogated caffeine (0.5 mM) induced decreases in peak inward (57.27 ± 2.8 pA/μm^2^ vs. 52.47 ± 3.31 pA/μm^2^, *p* = 0.6117, n = 10) current in agreement with its action in SkM (Liu et al., 2021). Maximum (105.61 ± 5.23 vs. 99.60 ± 5.37 pA/μm^2^, *p* = 0.9185), transient outward (60.11 ± 3.87 vs. 53.96 ± 5.47 pA/μm^2^, *p* = 0.5576), and prolonged outward currents were now similarly left unchanged (45.5 ± 2.93 vs. 45.64 ± 2.47 pA/μm^2^, *p* = 0.6164). Finally, a 2 mM caffeine challenge in the presence of CPA did not affect inward (60.08 ± 7.29 vs. 64.75 ± 7.07 pA/μm^2^, *p* = 0.8266, n = 5) or maximum (104.55 ± 6.43 vs. 102.58 ± 8.39 pA/μm^2^, *p* = 0.9768) and transient outward current components (61.45 ± 6.45 vs. 49.79 ± 6.5 pA/μm^2^, *p* = 0.2848) (Figure 8). Caffeine continued to increase the prolonged outward current (43.1 ± 1.54 vs. 52.79 ± 2.03 pA/μm^2^, *p* = 0.0009).

## 4 Discussion

Intracellular Ca^2+^ homeostasis has been extensively studied in connection with skeletal (SkM) and cardiac muscle excitation–contraction coupling. In this process, Nav1.4- or Nav1.5-mediated action potential generation (Adrian and Peachey, 1973) elevates [Ca^2+^]_i_ from the low background ∼100 nM to contractile activating levels through RyR1- or RyR2-mediated SR Ca^2+^ store release (Chawla et al., 2001). The latter involves direct allosteric or Ca^2+^-induced coupling following Cav1.1- or Cav1.2-mediated voltage-sensing or Ca^2+^-entry (Huang et al., 2011). The action was localized to potential Ca^2+^ domains within triad or dyad (T-SR) junctions between surface tubular and SR membranes (Bardsley et al., 2021).

Ca^2+^ homeostasis is similarly central to neuronal physiology. In hippocampal CA1 pyramidal neurons, it may modulate cell excitability and synaptic function clinically implicated in age-related cognitive decline (Mattson and Chan, 2003; Foster, 2007; Kumar et al., 2009; Burke and Barnes, 2010; Oh et al., 2010), extending to cell death (Stanika et al., 2012). In common with skeletal and cardiac muscles, CA1 pyramidal neurons possess voltage-gated Na^+^, Ca^2+^, and K^+^, as well as RyR-Ca^2+^ release channels and endoplasmic reticular (ER)–plasma membrane (PM) junctions (EPJs). However, they contain different Nav, Cav, Kv, and RyR subtypes. Anti-RyR antibody labeling methods (Furuichi et al., 1994; Giannini et al., 1995) suggest the expression of all three established RyR1-3 subtypes (McPherson and Campbell, 1993). Studies in RyR3^−/−^ mouse CA1 cells implicate highly expressed RyR3 potentiating slow afterhyperpolarizing current, sIAHP. Murine models expressing GFP-tagged RyR2 demonstrated significant expression of RyR2 in CA1 pyramidal neurons (Hiess et al., 2022). They show Cav1.3–RyR3 interactions promoted by Kv2.1 within Kv2.1-LTCC-RyR triads (Johnson et al., 2018; Vierra et al., 2019) and interactions between Cav3.1 and Cav1.2 and Kv4.2 in hippocampal dendrites and between the resulting altered intracellular Ca^2+^ and K_Ca_ late in action potential membrane repolarization (Verkhratsky and Shmigol, 1996; Vergara et al., 1998). Their parallel increases and decreases in amplitude suggested interactions between Na^+^ influx and transient outward *I*_K_ in intact neurons (Marrero and Lemos, 2003), resembling features reported of a further K^+^ channel subtype (Bhattacharjee and Kaczmarek, 2005; Hage and Salkoff, 2012). Finally, RyR activity could influence hippocampal neuronal firing, overall activity (Tedoldi et al., 2020), and synaptic transmission and plasticity (Johenning et al., 2015). RyR dysregulation has been implicated in long-term potentiation and depression (Maffei, 2018), key to hippocampal memory formation (Hiess et al., 2022). It has been linked to a wide range of neurological disorders, ranging from epilepsy (Lehnart et al., 2008) to Alzheimer’s disease (Chiantia et al., 2023).

Recent reports described the feedback effects of both background and evoked RyR-mediated intracellular store Ca^2+^ release upon Na^+^ channel function in intact loose-patch clamped skeletal and cardiac myocytes. These findings fulfilled predictions of structural evidence for either direct or Ca^2+^ calmodulin-mediated Ca^2+^ action on Nav C-terminal domains (Salvage et al., 2021; Salvage et al., 2023). Caffeine at 0.5 and 2 mM, respectively, decreased and increased *I*_Na_. This was consistent with its RyR agonist action at low (0.5 mM) and ultimate RyR inhibitory effects at high (>1.0 mM) concentrations. In the latter situation, caffeine induces a rapid transient [Ca^2+^]_i_ increase followed by its decrease below even resting levels, attributed to a combination of an initial SERCA activation and subsequent sustained RyR inactivation (See Introduction; (Pagala and Taylor, 1998; Vogalis et al., 2001)).

The RyR blocker dantrolene and SERCA inhibitor cyclopiazonic acid then themselves increased *I*_Na_, suggesting the effects of a reduced background Ca^2+^ on *I*_Na_. These findings fulfilled expectations from actions on *I*_Na_ of blocking RyR-mediated Ca^2+^ release (Sarbjit-Singh et al., 2020) or of ER Ca^2+^ store depletion by SERCA pump inhibition (Seidler et al., 1989; Liu et al., 2021). Both actions would reduce the resting release of SR store Ca^2+^, increasing the background [Ca^2+^] within a T-SR Ca^2+^ domain (Bardsley et al., 2021). These agents also abrogated caffeine actions when the agents were applied in combination, consistent with common actions on SR store Ca^2+^ release (Matthews et al., 2019; Sarbjit-Singh et al., 2020; Liu et al., 2021). Together, these findings suggested the existence of inhibitory feedback effects of released Ca^2+^ upon Na^+^ channel function and that this took place within microdomains of elevated [Ca^2+^] within the T-SR junctions. Here, resting [Ca^2+^]_i_ was both elevated by a background RyR-mediated Ca^2+^ release and further elevated by RyR-agonists (Bardsley et al., 2021).

We here investigate parallel effects within native *in situ* CA1 pyramidal neurons in murine hippocampal coronal slices using similar pharmacological manipulations. Continued use of a loose-patch clamping approach permitted the study of ionic currents using successive low-resistance seals, leaving cell surface membranes intact and changes in intracellular Ca^2+^ homeostasis unperturbed before and after pharmacological challenges (Matthews et al., 2019; Sarbjit-Singh et al., 2020; Liu et al., 2021). This loose-patch clamping involves specific circuitry required to correct for the much greater leaks and consequent pipette currents. Thus, the true membrane current is much smaller than the leak current, which must be corrected before feeding into the actual patch clamp circuit. Such corrections are potentially limited by the ability of the Ag/AgCl junction for sustained current delivery through the loose-patch electrode. Nevertheless, the approach permits effective overall assessments for, and examination of, current contributions and their comparison before and following physiological interventions in intact cells without disrupting intracellular contents. Conventional high-resistance tight-patch seals can contrastingly involve membrane disruption and possible alterations in [Ca^2+^]_i_ by the use of pipettes containing Ca^2+^-sequestrating ethylene glycol-bis (β-aminoethyl ether)-N, N, N′, N′-tetra acetic acid with its own Ca^2+^ buffering capacity.

The loose-patch recordings demonstrated transient inward followed by transient, then prolonged outward currents in response to depolarizing steps, agreeing with previous reports with the same technique in other neuronal cell types that had *I*_Na_ and *I*_K_ in these deflections (Marrero and Lemos, 2003). As in the previous reports, Na^+^ replacement experiments abolished peak inward and reduced transient outward currents. In the latter respect, these features resembled those of outward Na^+^-activated K^+^ (Slack and Slick, KCNT1 and KCNT2) currents (Bhattacharjee and Kaczmarek, 2005; Hage and Salkoff, 2012). They did not affect prolonged outward currents. Further controls successively used both low (10 μM) and high nifedipine concentrations (100 μM), respectively, selectively or non-selectively blocking high and both high- and low-voltage-activated Ca^2+^ channel activity (Curtis and Scholfield, 2001; Perez-Reyes, 2003). Neither blocking conditions affected either inward, or transient or prolonged outward, current. Thus, the inward currents did not include detectable Cav contributions. These controls also exclude regulatory contributions attributable to any associated influxes of extracellular Ca^2+^ upon the observed inward and outward currents.

The experiments then explored the effects on voltage-gated membrane currents of manipulations directed instead at RyR-mediated release of SR store Ca^2+^, using protocols that directly paralleled the similar loose-patch clamp *I*_Na_ investigations in SkM. These findings, for the first time, suggest a similarity between hippocampal CA1 pyramidal neurons and skeletal and cardiac myocytes in that caffeine-induced increases or decreases in RyR-mediated ER Ca^2+^ release either decreased or increased inward current. Consistent with previous observations, they also demonstrate accompanying changes in transient but not prolonged outward current. Here, low (0.5 mM) RyR-agonist caffeine concentrations, expected to increase RyR-mediated store Ca^2+^ release into the cytosol, reduced both inward and transient outward currents, resembling previously reported *I*_Na_ alterations in SkM (Sarbjit-Singh et al., 2020; Liu et al., 2021), as well as previously suggested interactions between inward *I*_Na_ and transient outward *I*_K_ in intact neurons (Marrero and Lemos, 2003). The prolonged outward currents contrastingly remained unchanged. Such a mechanism could provide negative regulatory feedback on Nav activity, following excessive ER Ca^2+^ release in murine CA1 pyramidal neurons.

In other respects, results in CA1 pyramidal neurons contrasted with SkM findings. First, a higher (2 mM) caffeine concentration, instead of increasing inward current, left both inward and transient outward currents unchanged. Second, applications of the respective RyR and SERCA blockers dantrolene (10 μM) and CPA (1 μM) by themselves, in contrast to previous SkM findings (Sarbjit-Singh et al., 2020; Liu et al., 2021), altered neither inward nor outward currents. Third, both dantrolene and CPA, nevertheless, reversed the effects of caffeine on peak inward current when either of these agents was given in combination with caffeine. In agreement with previous findings in SkM, following pretreatment, either dantrolene or CPA applied in combination abrogated the effects of both 0.5 mM and 2 mM caffeine, leaving inward and outward currents indistinguishable from control results obtained in the absence of any pharmacological agent. This was consistent with all three agents acting directly or indirectly on the ER store Ca^2+^ release. In considering the possible involvement of specific RyR isoforms in these findings, recent *in vitro* reports provide evidence that dantrolene acts on RyR1 and RyR3 but not RyR2 (Zhao et al., 2001). However, this contrasts with its effective *in vivo* action with anti-arrhythmic consequences in cardiac cells, known to express RyR2 (Kobayashi et al., 2009; Kobayashi et al., 2010; Avula et al., 2018; Greco et al., 2022). Previous reports also indicate that it abrogates the effects of pharmacological challenge on *I*_Na_ (Salvage et al., 2015; Li et al., 2017; Valli et al., 2018b) and arrhythmic phenotype in intact cardiac tissue (Hartmann et al., 2017; Nofi et al., 2020). The present findings themselves also describe its abrogation of the effect of caffeine challenge in reducing *I*_Na_. Finally, besides interactions between Ca^2+^ and delayed rectifier Kvs (Misonou et al., 2005), CA1 pyramidal cells possess intermediate-conductance Ca^2+^-dependent K^+^ (*I*_KCa_) channels whose activation by intracellular [Ca^2+^] elevation by membrane Cav action is amplified by RyR-mediated store Ca^2+^ release (Verkhratsky and Shmigol, 1996; Vergara et al., 1998). The latter may underlie slow afterhyperpolarization (sAHP), following trains of synaptic input or postsynaptic stimuli (King et al., 2015; Tedoldi et al., 2020).

However, such sAHP strongly contributes to the prolonged outward current component, reaching maximum amplitude over longer time courses of several hundred milliseconds (Lancaster and Adams, 1986). Nevertheless, such mechanisms could complement established longer timescale, similarly Ca^2+^-related, K^+^ channel regulation in skeletal (Maqoud et al., 2017) and cardiac muscles (Yang et al., 2022; Liu et al., 2023).

Both the present and previously reported studies were made in, and were, therefore, applicable to, intact CA1 and skeletal muscle cells with unperturbed Ca^2+^ homeostatic and surface membrane mechanisms. This involved the applied investigational agents partitioning across cell surface membranes to access their respective intracellular targets. In addition, larger RyR-mediated Ca^2+^ elevations could additionally act on inhibitory RyR Ca^2+^ binding sites, giving the previously reported bimodal [Ca^2+^]_i_ effects (Laver and Curtis, 1996; Pagala and Taylor, 1998; Vogalis et al., 2001) and be affected by cytosolic Ca^2+^ buffering and SERCA-mediated Ca^2+^ SR reuptake. Such a scheme was corroborated by comparing results from three separate agents, namely, caffeine, dantrolene, and CPA, each directed at distinct aspects of Ca^2+^ homeostasis. Together, the findings thus suggest, for the first time, the direct effects of ER-mediated modulation of intracellular calcium on inward and transient outward surface membrane currents. They merit future explorations as to whether the present findings reflect a potential non-canonical mechanism regulating membrane excitability through actions on Nav triggering or the resulting neuronal firing rates. The loose-patch technique could also be used to investigate voltage-gated ionic currents and their modulation not only in the cell body but also in the dendrites of hippocampal neurons. This could provide information about the functional compartmentalization of Nav and Kv channel cellular functions. Finally, the technique could be extended to follow successive stages of development in animals at different age groups.

## Data Availability

The original contributions presented in the study are included in the article/Supplementary Material; further inquiries can be directed to the corresponding authors.

## References

[B1] Adrian R. Peachey L. (1973). Reconstruction of the action potential of frog sartorius muscle. J. physiology 235, 103–131. 10.1113/jphysiol.1973.sp010380 PMC1350735 4778131

[B2] Ahmad S. Valli H. Smyth R. Jiang A. Y. Jeevaratnam K. Matthews H. R. (2019). Reduced cardiomyocyte Na+ current in the age-dependent murine Pgc-1β-/- model of ventricular arrhythmia. J. Cell. physiology 234, 3921–3932. 10.1002/jcp.27183 PMC6492124 30146680

[B3] Almers W. Stanfield P. Stühmer W. (1983). Lateral distribution of sodium and potassium channels in frog skeletal muscle: measurements with a patch‐clamp technique. J. physiology 336, 261–284. 10.1113/jphysiol.1983.sp014580 PMC1198969 6308223

[B4] Anderson D. Rehak R. Hameed S. Mehaffey W. H. Zamponi G. W. Turner R. W. (2010). Regulation of the KV4. 2 complex by CaV3. 1 calcium channels. Channels 4, 163–167. 10.4161/chan.4.3.11955 20458163

[B5] Armstrong C. Matteson D. (1985). Two distinct populations of calcium channels in a clonal line of pituitary cells. Science 227, 65–67. 10.1126/science.2578071 2578071

[B6] Avula U. M. R. Hernandez J. J. Yamazaki M. Valdivia C. R. Chu A. Rojas-Pena A. (2018). Atrial infarction-induced spontaneous focal discharges and atrial fibrillation in sheep: role of dantrolene-sensitive aberrant ryanodine receptor calcium release. Circulation Arrhythmia Electrophysiol. 11, e005659. 10.1161/CIRCEP.117.005659 PMC6554725 29540372

[B7] Bardsley O. J. Matthews H. R. Huang C. L.-H. (2021). Finite element analysis predicts Ca2+ microdomains within tubular-sarcoplasmic reticular junctions of amphibian skeletal muscle. Sci. Rep. 11, 14376. 10.1038/s41598-021-93083-1 34257321 PMC8277803

[B8] Berridge M. J. (1998). Neuronal calcium signaling. Neuron 21, 13–26. 10.1016/s0896-6273(00)80510-3 9697848

[B9] Bhattacharjee A. Kaczmarek L. K. (2005). For K+ channels, Na+ is the new Ca2+. Trends Neurosci. 28, 422–428. 10.1016/j.tins.2005.06.003 15979166

[B10] Burke S. N. Barnes C. A. (2010). Senescent synapses and hippocampal circuit dynamics. Trends Neurosci. 33, 153–161. 10.1016/j.tins.2009.12.003 20071039 PMC3076741

[B11] Chawla S. Skepper J. N. Hockaday A. R. Huang C. L.-H. (2001). Calcium waves induced by hypertonic solutions in intact frog skeletal muscle fibres. J. Physiology 536, 351–359. 10.1111/j.1469-7793.2001.0351c.xd PMC2278869 11600671

[B12] Chiantia G. Hidisoglu E. Marcantoni A. (2023). The role of ryanodine receptors in regulating neuronal activity and its connection to the development of Alzheimer’s disease. Cells 12, 1236. 10.3390/cells12091236 37174636 PMC10177020

[B91] Chin J. Y. Matthews H. R. Fraser J. A. Skepper J. N. Chawla S. Huang C. L. H. (2004). Detubulation experiments localise delayed rectifier currents to the surface membrane of amphibian skeletal muscle fibres. J. Muscle Res. Cell Motil. 25, 389–395. 10.1007/s10947-004-4069-9 15548868

[B13] Clapham D. E. (2007). Calcium signaling. Cell 131, 1047–1058. 10.1016/j.cell.2007.11.028 18083096

[B14] Curtis T. M. Scholfield C. N. (2001). Nifedipine blocks Ca2+ store refilling through a pathway not involving L‐type Ca2+ channels in rabbit arteriolar smooth muscle. J. physiology 532, 609–623. 10.1111/j.1469-7793.2001.0609e.x PMC2278590 11313433

[B15] Dettbarn C. Györke S. Palade P. (1994). Many agonists induce" quantal" Ca2+ release or adaptive behavior in muscle ryanodine receptors. Mol. Pharmacol. 46, 502–507. 7935331

[B16] Ellis K. Castellion A. Honkomp L. Wessels F. Carpenter J. Halliday R. (1973). Dantrolene, a direct acting skeletal muscle relaxant. J. Pharm. Sci. 62, 948–951. 10.1002/jps.2600620619 4712630

[B17] Foster T. C. (2007). Calcium homeostasis and modulation of synaptic plasticity in the aged brain. Aging Cell 6, 319–325. 10.1111/j.1474-9726.2007.00283.x 17517041

[B18] French C. Sah P. Buckett K. Gage P. (1990). A voltage-dependent persistent sodium current in mammalian hippocampal neurons. J. General Physiology 95, 1139–1157. 10.1085/jgp.95.6.1139 PMC2216358 2374000

[B19] Fryer M. Neering I. (1989). Actions of caffeine on fast‐and slow‐twitch muscles of the rat. J. physiology 416, 435–454. 10.1113/jphysiol.1989.sp017770 PMC1189224 2607458

[B20] Furuichi T. Furutama D. Hakamata Y. Nakai J. Takeshima H. Mikoshiba K. (1994). Multiple types of ryanodine receptor/Ca2+ release channels are differentially expressed in rabbit brain. J. Neurosci. 14, 4794–4805. 10.1523/JNEUROSCI.14-08-04794.1994 8046450 PMC6577160

[B21] Ghasemi Z. Naderi N. Shojaei A. Raoufy M. R. Ahmadirad N. Mirnajafi-Zadeh J. (2018). Effect of low-frequency electrical stimulation on the high-K+-induced neuronal hyperexcitability in rat hippocampal slices. Neuroscience 369, 87–96. 10.1016/j.neuroscience.2017.11.012 29138107

[B22] Giannini G. Conti A. Mammarella S. Scrobogna M. Sorrentino V. (1995). The ryanodine receptor/calcium channel genes are widely and differentially expressed in murine brain and peripheral tissues. J. Cell Biol. 128, 893–904. 10.1083/jcb.128.5.893 7876312 PMC2120385

[B23] Greco L. V. Migirov A. Ojamaa K. Li Y. Huang Y. Kobayashi S. (2022). Stabilizing cardiac ryanodine receptor with dantrolene treatment prevents binge alcohol–enhanced atrial fibrillation in rats. J. Cardiovasc. Pharmacol. 80, 739–745. 10.1097/FJC.0000000000001346 35947104

[B24] Hage T. A. Salkoff L. (2012). Sodium-activated potassium channels are functionally coupled to persistent sodium currents. J. Neurosci. 32, 2714–2721. 10.1523/JNEUROSCI.5088-11.2012 22357855 PMC3319674

[B25] Hartmann N. Pabel S. Herting J. Schatter F. Renner A. Gummert J. (2017). Antiarrhythmic effects of dantrolene in human diseased cardiomyocytes. Heart Rhythm. 14, 412–419. 10.1016/j.hrthm.2016.09.014 27650424

[B26] Head S. (1993). Membrane potential, resting calcium and calcium transients in isolated muscle fibres from normal and dystrophic mice. J. physiology 469, 11–19. 10.1113/jphysiol.1993.sp019801 PMC1143858 8271194

[B27] Herrmann-Frank A. Lüttgau H.-C. George S. D. (1999). Caffeine and excitation–contraction coupling in skeletal muscle: a stimulating story. J. Muscle Res. Cell Motil. 20, 223–236. 10.1023/a:1005496708505 10412093

[B28] Hiess F. Yao J. Song Z. Sun B. Zhang Z. Huang J. (2022). Subcellular localization of hippocampal ryanodine receptor 2 and its role in neuronal excitability and memory. Commun. Biol. 5, 183. 10.1038/s42003-022-03124-2 35233070 PMC8888588

[B29] Huang C. L.-H. Pedersen T. H. Fraser J. A. (2011). Reciprocal dihydropyridine and ryanodine receptor interactions in skeletal muscle activation. J. muscle Res. Cell Motil. 32, 171–202. 10.1007/s10974-011-9262-9 21993921

[B30] Johenning F. W. Theis A.-K. Pannasch U. Rückl M. Rüdiger S. Schmitz D. (2015). Ryanodine receptor activation induces long-term plasticity of spine calcium dynamics. PLoS Biol. 13, e1002181. 10.1371/journal.pbio.1002181 26098891 PMC4476683

[B31] Johnson B. Leek A. N. Solé L. Maverick E. E. Levine T. P. Tamkun M. M. (2018). Kv2 potassium channels form endoplasmic reticulum/plasma membrane junctions via interaction with VAPA and VAPB. Proc. Natl. Acad. Sci. 115, E7331–E7340. 10.1073/pnas.1805757115 29941597 PMC6077746

[B32] King B. Rizwan A. P. Asmara H. Heath N. C. Engbers J. D. Dykstra S. (2015). IKCa channels are a critical determinant of the slow AHP in CA1 pyramidal neurons. Cell Rep. 11, 175–182. 10.1016/j.celrep.2015.03.026 25865881

[B33] Klee R. Ficker E. Heinemann U. (1995). Comparison of voltage-dependent potassium currents in rat pyramidal neurons acutely isolated from hippocampal regions CA1 and CA3. J. neurophysiology 74, 1982–1995. 10.1152/jn.1995.74.5.1982 8592191

[B34] Kobayashi S. Yano M. Suetomi T. Ono M. Tateishi H. Mochizuki M. (2009). Dantrolene, a therapeutic agent for malignant hyperthermia, markedly improves the function of failing cardiomyocytes by stabilizing interdomain interactions within the ryanodine receptor. J. Am. Coll. Cardiol. 53, 1993–2005. 10.1016/j.jacc.2009.01.065 19460614 PMC2764410

[B35] Kobayashi S. Yano M. Uchinoumi H. Suetomi T. Susa T. Ono M. (2010). Dantrolene, a therapeutic agent for malignant hyperthermia, inhibits catecholaminergic polymorphic ventricular tachycardia in a RyR2R2474S/+ knock-in mouse model. Circulation J. 74, 2579–2584. 10.1253/circj.cj-10-0680 20944434

[B36] Kodirov S. A. (2023). Whole-cell patch-clamp recording and parameters. Biophys. Rev. 15, 257–288. 10.1007/s12551-023-01055-8 37124922 PMC10133435

[B37] Kumar A. Bodhinathan K. Foster T. C. (2009). Susceptibility to calcium dysregulation during brain aging. Front. aging Neurosci. 2, 3389. 10.3389/neuro.24.002.2009 PMC2874411 20552053

[B38] Lancaster B. Adams P. (1986). Calcium-dependent current generating the afterhyperpolarization of hippocampal neurons. J. neurophysiology 55, 1268–1282. 10.1152/jn.1986.55.6.1268 2426421

[B39] Laver D. R. Curtis B. A. (1996). Response of ryanodine receptor channels to Ca2+ steps produced by rapid solution exchange. Biophysical J. 71, 732–741. 10.1016/S0006-3495(96)79272-X PMC1233529 8842211

[B40] Leech C. A. Holz I. V. G. G. (1994). Application of patch clamp methods to the study of calcium currents and calcium channels. Methods Cell Biol. 40, 135–151. 10.1016/s0091-679x(08)61113-9 8201974 PMC3509330

[B41] Lehnart S. E. Mongillo M. Bellinger A. Lindegger N. Chen B.-X. Hsueh W. (2008). Leaky Ca 2+ release channel/ryanodine receptor 2 causes seizures and sudden cardiac death in mice. J. Clin. investigation 118, 2230–2245. 10.1172/JCI35346 PMC2381750 18483626

[B42] Li M. Hothi S. S. Salvage S. C. Jeevaratnam K. Grace A. A. Huang C. L. H. (2017). Arrhythmic effects of Epac‐mediated ryanodine receptor activation in Langendorff‐perfused murine hearts are associated with reduced conduction velocity. Clin. Exp. Pharmacol. Physiology 44, 686–692. 10.1111/1440-1681.12751 PMC5488224 28316073

[B43] Liang L. Wei H. (2015). Dantrolene, A treatment for alzheimer's disease? Alzheimer Dis. Assoc. Disord. 29, 1–5. 10.1097/WAD.0000000000000076 25551862 PMC4334699

[B44] Liu S. X. Matthews H. R. Huang C. L.-H. (2021). Sarcoplasmic reticular Ca2+-ATPase inhibition paradoxically upregulates murine skeletal muscle Nav1. 4 function. Sci. Rep. 11, 2846. 10.1038/s41598-021-82493-w 33531589 PMC7854688

[B45] Liu T. Li T. Xu D. Wang Y. Zhou Y. Wan J. (2023). Small-conductance calcium-activated potassium channels in the heart: expression, regulation and pathological implications. Philosophical Trans. R. Soc. B 378, 20220171. 10.1098/rstb.2022.0171 PMC10150224 37122223

[B46] Liu Z. Li L. Liu C. (2001). Blind patch clamp whole-cell recording technique for neurons in hippocampal slices. Sheng li xue bao:Acta Physiol. Sin. 53, 405–408. 11833428

[B47] MacGregor D. G. Avshalumov M. V. Rice M. E. (2003). Brain edema induced by *in vitro* ischemia: causal factors and neuroprotection. J. Neurochem. 85, 1402–1411. 10.1046/j.1471-4159.2003.01772.x 12787060

[B48] Maffei A. (2018). Long-term potentiation and long-term depression. Oxford research encyclopedia of neuroscience.

[B49] Magee J. C. Johnston D. (1995). Characterization of single voltage‐gated Na+ and Ca2+ channels in apical dendrites of rat CA1 pyramidal neurons. J. physiology 487, 67–90. 10.1113/jphysiol.1995.sp020862 PMC1156600 7473260

[B50] Maqoud F. Cetrone M. Mele A. Tricarico D. (2017). Molecular structure and function of big calcium-activated potassium channels in skeletal muscle: pharmacological perspectives. Physiol. genomics 49, 306–317. 10.1152/physiolgenomics.00121.2016 28455309

[B51] Marrero H. G. Lemos J. R. (2003). Loose-patch clamp currents from the hypothalamo-neurohypophysial system of the rat. Pflügers Arch. 446, 702–713. 10.1007/s00424-003-1120-1 12898256

[B52] Martin C. A. Petousi N. Chawla S. Hockaday A. R. Burgess A. J. Fraser J. A. (2003). The effect of extracellular tonicity on the anatomy of triad complexes in amphibian skeletal muscle. J. Muscle Res. Cell Motil. 24, 407–415. 10.1023/a:1027356410698 14677643

[B53] Martina M. Schultz J. H. Ehmke H. Monyer H. Jonas P. (1998). Functional and molecular differences between voltage-gated K+ channels of fast-spiking interneurons and pyramidal neurons of rat hippocampus. J. Neurosci. 18, 8111–8125. 10.1523/JNEUROSCI.18-20-08111.1998 9763458 PMC6792860

[B54] Mathias R. T. Cohen I. S. Oliva C. (1990). Limitations of the whole cell patch clamp technique in the control of intracellular concentrations. Biophysical J. 58, 759–770. 10.1016/S0006-3495(90)82418-8 PMC1281016 2169920

[B55] Matthews H. R. Tan S. R. Shoesmith J. A. Ahmad S. Valli H. Jeevaratnam K. (2019). Sodium current inhibition following stimulation of exchange protein directly activated by cyclic-3′, 5′-adenosine monophosphate (Epac) in murine skeletal muscle. Sci. Rep. 9, 1927–2013. 10.1038/s41598-018-36386-0 30760734 PMC6374420

[B56] Mattson M. P. Chan S. L. (2003). Neuronal and glial calcium signaling in Alzheimer’s disease. Cell calcium 34, 385–397. 10.1016/s0143-4160(03)00128-3 12909083

[B57] McPherson P. Campbell K. (1993). The ryanodine receptor/Ca2+ release channel. J. Biol. Chem. 268, 13765–13768. 10.1016/s0021-9258(19)85166-9 8390976

[B58] Milton R. Caldwell J. (1990). How do patch clamp seals form? A lipid bleb model. Pflügers Arch. 416, 758–762. 10.1007/BF00370626 1701047

[B59] Minlebaev M. Valeeva G. Tcheremiskine V. Coustillier G. Khazipov R. (2013). Cell-attached recordings of responses evoked by photorelease of GABA in the immature cortical neurons. Front. Cell. Neurosci. 7, 83. 10.3389/fncel.2013.00083 23754981 PMC3668178

[B60] Misonou H. Mohapatra D. P. Menegola M. Trimmer J. S. (2005). Calcium-and metabolic state-dependent modulation of the voltage-dependent Kv2. 1 channel regulates neuronal excitability in response to ischemia. J. Neurosci. 25, 11184–11193. 10.1523/JNEUROSCI.3370-05.2005 16319318 PMC6725654

[B61] Murphy J. G. Gutzmann J. J. Lin L. Hu J. Petralia R. S. Wang Y.-X. (2022). R-type voltage-gated Ca2+ channels mediate A-type K+ current regulation of synaptic input in hippocampal dendrites. Cell Rep. 38, 110264. 10.1016/j.celrep.2021.110264 35045307 PMC10496648

[B62] Nofi C. Zhang K. Tang Y.-D. Li Y. Migirov A. Ojamaa K. (2020). Chronic dantrolene treatment attenuates cardiac dysfunction and reduces atrial fibrillation inducibility in a rat myocardial infarction heart failure model. Heart Rhythm. 1, 126–135. 10.1016/j.hroo.2020.03.004 PMC8183840 34113867

[B63] Oh M. M. Oliveira F. A. Disterhoft J. F. (2010). Learning and aging related changes in intrinsic neuronal excitability. Front. aging Neurosci. 2, 2. 10.3389/neuro.24.002.2010 20552042 PMC2874400

[B64] Pagala M. K. Taylor S. R. (1998). Imaging caffeine-induced Ca2+ transients in individual fast-twitch and slow-twitch rat skeletal muscle fibers. Am. J. Physiology-Cell Physiology 274, C623–C632. 10.1152/ajpcell.1998.274.3.C623 9530093

[B65] Perez-Reyes E. (2003). Molecular physiology of low-voltage-activated t-type calcium channels. Physiol. Rev. 83, 117–161. 10.1152/physrev.00018.2002 12506128

[B66] Perkins K. L. (2006). Cell-attached voltage-clamp and current-clamp recording and stimulation techniques in brain slices. J. Neurosci. methods 154, 1–18. 10.1016/j.jneumeth.2006.02.010 16554092 PMC2373773

[B67] Roberts W. Stühmer W. Weiss R. Stanfield P. Almers W. (1986). Distribution and mobility of voltage-gated ion channels in skeletal muscle. Ann. N. Y. Acad. Sci. 479, 377–384. 10.1111/j.1749-6632.1986.tb15583.x 2434003

[B68] Roberts W. M. Almers W. (1992). Patch voltage clamping with low-resistance seals: loose patch clamp. Methods Enzym. 207, 155–176. 10.1016/0076-6879(92)07011-c 1382182

[B69] Salvage S. King J. Chandrasekharan K. Jafferji D. Guzadhur L. Matthews H. (2015). Flecainide exerts paradoxical effects on sodium currents and atrial arrhythmia in murine R yR2‐P2328S hearts. Acta Physiol. 214, 361–375. 10.1111/apha.12505 PMC4510817 25850710

[B70] Salvage S. C. Dulhunty A. F. Jeevaratnam K. Jackson A. P. Huang C. L.-H. (2023). Feedback contributions to excitation–contraction coupling in native functioning striated muscle. Philosophical Trans. R. Soc. B 378, 20220162. 10.1098/rstb.2022.0162 PMC10150225 37122213

[B71] Salvage S. C. Habib Z. F. Matthews H. R. Jackson A. P. Huang C. L.-H. (2021). Ca2+-dependent modulation of voltage-gated myocyte sodium channels. Biochem. Soc. Trans. 49, 1941–1961. 10.1042/BST20200604 34643236 PMC8589445

[B72] Sandler V. M. Barbara J.-G. (1999). Calcium-induced calcium release contributes to action potential-evoked calcium transients in hippocampal CA1 pyramidal neurons. J. Neurosci. 19, 4325–4336. 10.1523/JNEUROSCI.19-11-04325.1999 10341236 PMC6782593

[B73] Sarbjit-Singh S. S. Matthews H. R. Huang C. L.-H. (2020). Ryanodine receptor modulation by caffeine challenge modifies Na+ current properties in intact murine skeletal muscle fibres. Sci. Rep. 10, 2199–2218. 10.1038/s41598-020-59196-9 32042141 PMC7010675

[B74] Seidler N. W. Jona I. Vegh M. Martonosi A. (1989). Cyclopiazonic acid is a specific inhibitor of the Ca2+-ATPase of sarcoplasmic reticulum. J. Biol. Chem. 264, 17816–17823. 10.1016/s0021-9258(19)84646-x 2530215

[B75] Stanika R. I. Villanueva I. Kazanina G. Andrews S. B. Pivovarova N. B. (2012). Comparative impact of voltage-gated calcium channels and NMDA receptors on mitochondria-mediated neuronal injury. J. Neurosci. 32, 6642–6650. 10.1523/JNEUROSCI.6008-11.2012 22573686 PMC3370824

[B76] Stühmer W. Almers W. (1982). Photobleaching through glass micropipettes: sodium channels without lateral mobility in the sarcolemma of frog skeletal muscle. Proc. Natl. Acad. Sci. 79, 946–950. 10.1073/pnas.79.3.946 6278504 PMC345870

[B77] Stühmer W. Roberts W. M. Almers W. (1983). The loose patch clamp. Single-channel recording. Spinger, 123–132.

[B78] Tedoldi A. Ludwig P. Fulgenzi G. Takeshima H. Pedarzani P. Stocker M. (2020). Calcium-induced calcium release and type 3 ryanodine receptors modulate the slow afterhyperpolarising current, sIAHP, and its potentiation in hippocampal pyramidal neurons. PLoS One 15, e0230465. 10.1371/journal.pone.0230465 32559219 PMC7304577

[B79] Ting J. T. Daigle T. L. Chen Q. Feng G. (2014). Acute brain slice methods for adult and aging animals: application of targeted patch clamp analysis and optogenetics. Patch-clamp methods Protoc. 1183, 221–242. 10.1007/978-1-4939-1096-0_14 PMC4219416 25023312

[B80] Valli H. Ahmad S. Jiang A. Y. Smyth R. Jeevaratnam K. Matthews H. R. (2018a). Cardiomyocyte ionic currents in intact young and aged murine Pgc-1β-/- atrial preparations. Mech. ageing Dev. 169, 1–9. 10.1016/j.mad.2017.11.016 29197478 PMC5846848

[B81] Valli H. Ahmad S. Sriharan S. Dean L. D. Grace A. A. Jeevaratnam K. (2018b). Epac‐induced ryanodine receptor type 2 activation inhibits sodium currents in atrial and ventricular murine cardiomyocytes. Clin. Exp. Pharmacol. Physiology 45, 278–292. 10.1111/1440-1681.12870 PMC5814738 29027245

[B82] Vazetdinova A. Valiullina-Rakhmatullina F. Rozov A. Evstifeev A. Khazipov R. Nasretdinov A. (2022). On the accuracy of cell-attached current-clamp recordings from cortical neurons. Front. Mol. Neurosci. 15, 979479. 10.3389/fnmol.2022.979479 36034500 PMC9405422

[B83] Vergara C. Latorre R. Marrion N. V. Adelman J. P. (1998). Calcium-activated potassium channels. Curr. Opin. Neurobiol. 8, 321–329. 10.1016/s0959-4388(98)80056-1 9687354

[B84] Verkhratsky A. Shmigol A. (1996). Calcium-induced calcium release in neurones. Cell calcium 19, 1–14. 10.1016/s0143-4160(96)90009-3 8653752

[B85] Vierra N. C. Kirmiz M. van der List D. Santana L. F. Trimmer J. S. (2019). Kv2. 1 mediates spatial and functional coupling of L-type calcium channels and ryanodine receptors in mammalian neurons. Elife 8, e49953. 10.7554/eLife.49953 31663850 PMC6839919

[B86] Vogalis F. Furness J. B. Kunze W. A. (2001). Afterhyperpolarization current in myenteric neurons of the Guinea pig duodenum. J. Neurophysiology 85, 1941–1951. 10.1152/jn.2001.85.5.1941 11353011

[B87] Wang C. Chung B. C. Yan H. Lee S.-Y. Pitt G. S. (2012). Crystal structure of the ternary complex of a NaV C-terminal domain, a fibroblast growth factor homologous factor, and calmodulin. Structure 20, 1167–1176. 10.1016/j.str.2012.05.001 22705208 PMC3610540

[B88] Xu N. Francis M. Cioffi D. L. Stevens T. (2014). Studies on the resolution of subcellular free calcium concentrations: a technological advance. Focus on "detection of differentially regulated subsarcolemmal calcium signals activated by vasoactive agonists in rat pulmonary artery smooth muscle cells". Am. Physiological Soc. 306, C636–C638. 10.1152/ajpcell.00046.2014 PMC3962594 24553184

[B89] Yan L. Fang Q. Zhang X. Huang B. (2020). Optimal pipette resistance, seal resistance, and zero-current membrane potential for loose patch or breakthrough whole-cell recording *in vivo* . Front. Neural Circuits 14, 34. 10.3389/fncir.2020.00034 32714153 PMC7344171

[B90] Yang B. Jiang Q. He S. Li T. Ou X. Chen T. (2022). Ventricular SK2 upregulation following angiotensin II challenge: modulation by p21-activated kinase-1. J. Mol. Cell. Cardiol. 164, 110–125. 10.1016/j.yjmcc.2021.11.001 34774547

[B92] Zhao F. Li P. Chen S. W. Louis C. F. Fruen B. R. (2001). Dantrolene inhibition of ryanodine receptor Ca2+ release channels: molecular mechanism and isoform selectivity. J. Biol. Chem. 276, 13810–13816. 10.1074/jbc.M006104200 11278295

